# Supplementary material for: AI applications in lumbar and lumbosacral pedicle screw placement: a systematic review of limited evidence and future directions
Source: Neurosurg Rev. 2026 Mar 17;49(1):283. doi: 10.1007/s10143-026-04192-2 (PMC12992485; doi:10.1007/s10143-026-04192-2)
Supplement: Supplementary file 1 — (DOCX 1.48 MB) [file 10143_2026_4192_MOESM1_ESM.docx]

**AI Applications in Lumbar and Lumbosacral Pedicle Screw Placement: A Systematic Review of Limited Evidence and Future Directions**

**Authors:**

Pakpoom Thintharua, ^1,2^ (pakpoom.thi@mahidol.ac.th)

Ratchaphon Prabrai, ^3^ (rp.prabrai@gmail.com)

Anuyut khamsiriwatchara ^4^ (anuyutk@gmail.com)

Rohan Sethi ^5^ (Rohsethi510@gmail.com)

Sorayouth Chumnanvej (Corresponding Author),^6^ (sorayouth.chu@mahidol.ac.th)

**Affiliations:**

1) Program in Translational Medicine, Faculty of Medicine Ramathibodi Hospital, Mahidol University, Samut Prakan, Thailand

2) Chakri Naruebodindra Medical Institute, Faculty of Medicine Ramathibodi Hospital, Mahidol University, Samut Prakan, Thailand

3) Airport of Thailand Public Company Limited, Bangkok, Thailand

4) Bachelor of Engineering (Biomedical Engineering), Faculty of Engineering, Royal Melbourne Institute of Technology University, Melbourne, Australia

5) Bachelor of Biological Sciences (Biomedical Sciences Module), Faculty of Science, Mahidol University International College, Nakhon Pathom, Thailand

6) Neurosurgery Division, Surgery Department, Faculty of Medicine Ramathibodi Hospital, Mahidol University, Bangkok, Thailand

**Abstract**

Artificial intelligence (AI) is a general term that refers to the use of a computer to simulate intelligent behavior with minimal human intervention. Currently, AI can be applied to various spine surgery approaches. This review aims to provide a clearer picture of AI's applicability for the perioperative period and enhance outcomes for pedicle screw fixation (PS). The PRISMA guideline was applied, which identified 14 studies regarding AI applications in PS. We categorized the AI application to PS into segmentation, object detection, image registration, and other categories, such as improved quality and converted images. Then, an analysis and discussion of the current trends and applications of various AI models in PS methods was performed. The effects of AI performance included a reduction in the time required for operations and planning, automatic identification of screws and anatomical landmarks, reduced image errors, and reduced radiation exposure. However, the lack of training data and less data diversity remain the limitations of model development, as both factors impact model generalization and robustness. This data extraction might reveal research gaps, providing researchers with ideas for future studies regarding AI and PS integration for better medical care outcomes.

**Keywords**

Artificial intelligence, medical imaging, pedicle screw fixation, spine surgery

**Introduction**

Artificial Intelligence (AI) lets computers act like people by analyzing data and making smart decisions. (1) It is transforming spine surgery by making it easier to assess patients before surgery and predict the postoperative outcomes. (2, 3) It is also improving the quality of medical research. (4) AI also makes surgical workflows and data tracking more efficient (2, 3) and directly helps with better performance during surgery. (2, 5) The medical image speed and quality analysis can be improved by AI while lowering cost. (4, 6) It also integrates robotics to assist—rather than replace—surgeons in delivering safer care. (3)

Pedicle screw fixation (PS) remains challenging, as placing screws near vital nerves involves significant risk. (5) Reports reveal that manual placement is difficult, with breach rates ranging from 8% to 50%. (7, 8) While the freehand technique is cost-effective, it relies heavily on the surgeon's experience and lacks real-time feedback. (9) To address these limitations, AI helps to improve image quality, automate trajectory planning, and enhance screw detection (11–15), making this error-prone process more efficient (10). Although wider studies on AI in spine surgery exist (2–4), there is currently no systematic review that focuses solely on AI applications for PS.

This is the first systematic review to examine current trends and applications of AI models in PS. Specifically, this systematic review aims to: (1) categorize current AI applications in pedicle screw fixation, (2) evaluate their performance metrics and clinical outcomes, (3) assess methodological quality, and (4) identify evidence gaps requiring future research. By synthesizing these findings, this study seeks to bridge the gap between technical innovation and clinical practice, helping surgeons select the optimal tools for safer outcomes.

**Materials and methods**

**Article collection**

The Preferred Reporting Items for Systematic Reviews and Meta-Analyses (PRISMA) guideline 2020 was utilized for the systematic review of literature. We searched the research articles based on three databases, including BASE: Pedicle Screws AND (Artificial Intelligence OR Neural Networks, Computer) AND Pedicle Screws AND (Radiography OR Magnetic Resonance Imaging) [January 15, 2025], PubMed: ((("Pedicle Screws"[Mesh]) AND ("Artificial Intelligence"[Mesh])) OR (("Pedicle Screws"[Mesh]) AND ("Neural Networks, Computer"[Mesh]))) AND ((("Pedicle Screws"[Mesh]) AND ("Radiography"[Mesh])) OR (("Pedicle Screws"[Mesh]) AND ("Magnetic Resonance Imaging"[Mesh]))) [January 15, 2025], and ScienceDirect: (("Pedicle Screws" AND "Artificial Intelligence") OR ("Pedicle Screws" AND "Neural Networks, Computer")) AND (("Pedicle Screws" AND "Radiography") OR ("Pedicle Screws" AND "Magnetic Resonance Imaging")) [January 16, 2025]. The articles were collected over ten years, from 2015 to 2025. The exclusion and inclusion criteria were set for selecting the appropriate research articles for references. The comprehensive search approach utilizing database-specific syntax is shown in Table S1 (Supplementary). The review focused on AI applications for pedicle screw fixation primarily in the lumbar and lumbosacral spine, as these represent the most common sites for pedicle screw instrumentation.

**Inclusion criteria**

This review article focuses on the application of AI models with pedicle screw fixation, which improves the surgical strategies. Articles should be written in the English language, full-length articles should be performed on human subjects, and articles should be published between 2015 and 2025. Furthermore, selected studies must have focused primarily on lumbar or lumbosacral pedicle screws, or on whole-spine applications where data for the lumbar/lumbosacral segments could be independently extracted.

**Exclusion criteria**

The types of articles, like review articles, case reports, letters to editors, only abstracts, paid articles, research on other procedures, and meta-analyses, were excluded from this literature. Articles without information regarding both pedicle screw fixation (PS) and its relationship with AI.

**Article screening process**

A two-stage screening process was used. Studies were first screened independently by two authors by title and abstract. Then, we performed an in-depth review of the selected articles to find any potentially eligible studies. Any disagreements at each stage were re-evaluated by two reviewers to reach a conclusive consensus.

**Data Extraction and Reporting**

Data from selected studies were extracted and compiled using Google Sheets (Google Inc., California, USA). Extracted variables included study characteristics (author, year, journal), methodological details (study design, population, sample size, perioperative phase, imaging modality), technical specifications (AI models, architectures), and key findings. Due to substantial heterogeneity in AI architectures, imaging modalities, outcome measures, and study designs, meta-analysis was not feasible. We conducted structured narrative synthesis using the PICO framework to systematically organize and compare findings. We organized and analyzed the data extracted using the PICO format. P: population (pedicle screws and spinal surgery), I: intervention (AI applications to pedicle screw fixation), C: comparison (AI and traditional approaches), and O: outcome (accuracy, precision, safety, and time-consuming).

**Quality Assessment**

All 14 studies were rated using the Quality Assessment of Diagnostic Accuracy Studies-2 (QUADAS-2) technique. Patient Selection, Index Test (AI model performance), Reference Standard (clinical grading systems), and Flow and Timing were examined by two authors for bias and applicability issues. A third reader was consulted if there was a disagreement; otherwise, judgments were determined by consensus.

**Statistical Analysis**

Cohen's Kappa (k) was used to measure the inter-rater reliability between the two separate reviewers. There was a lot of agreement (95.0% and a k-value of 0.89 in the first Stage 1 screening of the Title and Abstract. For the Full-Text review (Stage 2), dependability went up to a lot of agreement (k= 0.94; 97.8% agreement). All differences at both stages were worked out in a consensus discussion to make sure the final list of studies was correct.

**Results**

**Search Results**

The MeSH terminology was used to identify 1,492 articles in total that were searched from various online sources, including BASE (1,296), PubMed (53), and ScienceDirect (143), respectively. 1,168 articles remained after implementing the exclusion criteria, and 91 articles after using the inclusion criteria. From 91 articles, we selected only 14 for organization and analysis using the PICO format. We applied the systematic reviews and meta-analyses (PRISMA) for the study selection process, as shown in the flow diagram in **Fig. 1**.

All the selected articles were retrospective studies at 64.29% and prospective studies at 35.7%. Of the studies, 12 (85.7%) focused only on the lumbar or lumbosacral segment, whereas 2 (14.3%) included the whole spine but provided data relevant to the lumbar region. The perioperative period was observed, and it was found that the intraoperative phase was at 50.0%, followed by the preoperative phase at 28.6%, the postoperative phase at 14.3%, and both the pre- and intraoperative phases at 7.1%, respectively. The most common image modalities for AI applications in pedicle screw fixation were CT and CBCT scans at 35.7%, followed by radiography at 21.4%, the combination of at least two modalities at 21.4%, and MRI at 7.1%, respectively. In addition, 14.3% came from RGB or RGBD video sources and stereo camera images. The relevant characteristics of the included studies, including study designs, population, sample size, perioperative phase, image modalities, AI models, applications, and key findings, are presented in **Table 1**. Furthermore, what is the basic principle of each AI model, and why does it fit this task? was shown in **Table 2**.

Moreover, we found that 78.6% of the AI models included in the strategy were identifiable, whereas 21.43% were unidentified models, which referred to deep learning (DL) or machine learning (ML). The researchers selected architecture, which was a subset of DL, at a rate of 92.9%. U-Net-based architecture was the most prevalent DL model, representing 28.6%. Approximately 71.4% of articles effectively implemented their strategy with a singular model without integration with others. Furthermore, half of the overall research articles utilized AI models for image segmentation in comparison to other applications. We divided the successful application of AI models related to PS into four different categories, as illustrated in **Fig. 2**.


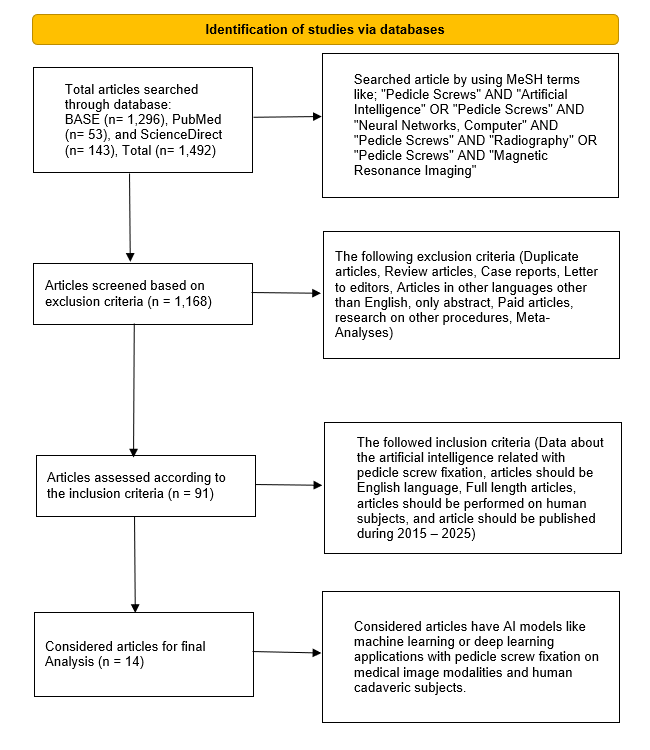


**Figure 1**. PRISMA flow diagram of systematic review

**Table 1.** Technical Performance and Clinical Analysis of AI Models in Spinal Surgery

| **Authors** | **Study design** | **Population** | **Sample size** | **Segments** | **Phase** | **Image modalities** | **AI models** | **Applications** | **Primary Metric** | **Performance Range** | **Clinical Significance** | **Study Limitations** |
| --- | --- | --- | --- | --- | --- | --- | --- | --- | --- | --- | --- | --- |
| Burström et al. 2019 (5) | PT | Human cadavers | 21 cadavers | Lumbar/  Lumbosacral | IO | CBCT | ML (unidentified) | Segmentation | Accuracy (Planning) | 86.1% (Auto) – 95.4% (Clinical) | Rapid automated planning (11 ± 4s per 5 vertebrae). | Performance relies on excluding severe deformities and prior surgeries. |
| Esfandiari et al. 2018 (9) | RT | Patients undergoing PS | 40 patients | Lumbar/  Lumbosacral | PO | X-rays | FCN | Segmentation | Segmentation Accuracy | 93% (Synthetic) vs. 83% (Clinical) | High precision in pose estimation (1.93° discrepancy). | Significant accuracy drops when moving from synthetic to clinical data. |
| Thies et al. 2020 (11) | RT | Chest CT scans from TCIA | n/a | Lumbar/  Lumbosacral | IO | CT, CBCT | Modified VGG architecture | Improved quality of the image | Image Quality | Qualitative improvement | Enables online C-arm trajectory adjustment without interruption. | Sample size and quantitative metrics not reported. |
| Siemionow et al. 2021 (12) | RT | Patients (unidentified) | 20 patients | Lumbar/  Lumbosacral | PrO, IO | CT | Autonomous pedicle screw planner (ML), CNN | Segmentation, landmark detection | Grading Agreement | 100% (Zdichavsky); 99% (Gertzbein Grade A) | Matches standard clinical grading systems (Ravi grading). | Small sample size (N=20). |
| Scherer et al. 2022 (13) | RT | Patients were derived from a consecutive registry of navigated spinal instrumentations | 179 patients | Lumbar/  Lumbosacral | IO | CT | nnU-Net | Segmentation | Mean Absolute Difference (MAD) | Screw tip MAD: ~4mm; Dice: 0.61 | 10x speed compared to manual planning. | Dice coefficient (0.61) suggests segmentation overlap is moderate. |
| Zhang et al. 2024 (14) | RT | Patients undergoing PS | 282 patients | Lumbar/  Lumbosacral | PrO | CT | Modified U-Net (combined with ResNet34) or VGG16 (limited data availability) architectures | Landmarks detection | Percentage of Correct Key points (PCK) | >93% (at 3mm threshold) | High inter-rater reliability (ICC 0.82–0.98) with clinicians. | Limited data availability noted for the VGG16 comparison arm. |
| Liebmann et al. 2024 (15) | PT | SpineDepth dataset and cadavers for validation | n/a (Dataset + Cadavers) | Whole-spine | IO | RGB or RGBD video sources | U-Net | Registration | Target Registration Error (TRE) | Median TRE: 2.7 mm; Success: 100% | Fast registration process (~1.5s total duration). | Exact sample size not fully defined in summary. |

**Table 1** (continued)

| **Authors** | **Study design** | **Population** | **Sample size** | **Segments** | **Phase** | **Image modalities** | **AI models** | **Applications** | **Primary Metric** | **Performance Range** | **Clinical Significance** | **Study Limitations** |
| --- | --- | --- | --- | --- | --- | --- | --- | --- | --- | --- | --- | --- |
| Yang et al. 2021 (16) | RT | The patients who had lumbar spine one-segment instrument surgery | 2894 lumbar spine | Lumbar/  Lumbosacral | PO | X-rays | ResNet34 model with ImageNet pretrained weights, Google AutoML, Apple Create ML | Object detection | Accuracy / Precision | ResNet: ~97-98%; AutoML: ~87-98% | Demonstrated high accuracy using transfer learning (ImageNet). | Commercial tool "Apple Create ML" showed significantly lower recall (73%). |
| von Atzigen et al. 2022 (17) | PT | Human cadavers | 8 cadavers | Lumbar/  Lumbosacral | IO | Stereo camera images | SNN | Object detection | Average Error / Time | 5.43 mm error; Bending time 231s | Marker-free AR reduced surgical bending time by ~50%. | Small cadaveric sample (N=8). |
| Roberts et al. 2023 (18) | RT | Patients who had CT and MRI scans performed within 30 days | 20 patients | Lumbar/  Lumbosacral | PrO | MRI | Supervised 3D cycleGan | Convert the type of image (MRI to CT) | Intraclass Correlation (ICC) | Sagittal error <10%; Axial error up to 34% | Enables CT-like planning from MRI (radiation-free). | High error rate (34%) in axial plane measurements; unreliable IVDH. |
| Abel et al. 2024 (19) | RT | Adult patients who planned for initial lumbar spine fusion surgery | 16 patients | Lumbar/  Lumbosacral | PrO | CT, MRI | DL (unidentified) | Segmentation | Geometric Reliability | High reliability (general) | Validates MRI usage for 3D geometric planning. | Failed to measure vertebral body length at L1, L2, and L4. |
| Luchmann et al. 2024 (20) | PT | Human cadavers | 6 cadavers | Lumbar/  Lumbosacral | IO | X-rays | DL (unidentified) | Segmentation | Breach Rate | 21% (AI) vs. 24% (Freehand) | Reduced radiation exposure (33 mGy vs. 49 mGy). | Very small sample (N=6); marginal improvement in breach rate. |
| Da Mutten et al. 2024 (21) | RT | Three different data sets from VerSe (spine), MSD T10 (liver), and COVID-19 (chest) | 214 patients for the training set and 40 CT scans for validation | Whole-spine | PrO | CT | YOLOv8m, 2D-U-Net | Object detection, segmentation | mAP / Dice Score | Dice: 0.76–0.79; mAP: 0.63 (Int) to 0.09 (Ext) | Segmentation generalized well to external validation. | Object detection failed to generalize externally (mAP dropped to 0.09). |
| Ao et al. 2025 (22) | PT | Adult humans with different BMIs | 5 adults | Lumbar/  Lumbosacral | IO | CT, MRI, US | SafeRPlan (Deep Reinforcement Learning) | Registration | Safety Rate | 99% Safety | Improved safety by 5% over existing methods. | Extremely small sample size (N=5). |

Augmented reality (AR), computed tomography (CT), cone beam computed tomography (CBCT), convolutional neural network (CNN), fully convolutional network (FCN), intervertebral disc height (IVDH), intraclass correlation coefficient (ICC), intraoperative (IO), machine learning (ML), mean average precision calculated at varying IoU thresholds (mAP), not available (n/a), pedicle screw fixation (PS), percentage of correct key points (PCK), postoperative (PO), preoperative (PrO), prospective study (PT), retrospective study (RT), stereo neural network (SNN), target registration error (TRE), the cancer imaging archive (TCIA), and ultrasound (US).

**Table 2**: Technical Principles of AI Models in Spinal Surgery

| **Model** | **Type** | **Core Working Principle (The "How")** | **Why does it fit this task?** |
| --- | --- | --- | --- |
| CNN (Convolutional Neural Network) | Foundational Architecture | Feature filtering: Small windows (filter) are moved over an image to find patterns. First, it finds lines or edges, then forms, and finally vertebrae. | It functions like human eyes, so the computer can "see" body shapes and features in CT or X-ray images instead of reading pixel values. |
| VGG16 | Deep CNN Backbone | Uniform Stacking: A very deep stack of simple 3*3 convolution filters. It relies on depth (16 layers) to learn increasingly complex features. | It's very simple: a consistent structure makes it a reliable "feature extractor" for improving image quality or detecting landmarks. |
| ResNet34 | Deep CNN Backbone | Residual Learning (Skip Connections): When networks are deep, data usually gets lost. ResNet adds layers with "skip connections" that let data go around certain levels. This keeps the original signal. | It can train much deeper networks (34 layers) without slowing down, which makes it a great tool for finding subtle fractures or features in X-rays. |
| ImageNet | Large-Scale Dataset | Transfer Learning: This is not a model, but a "school." Models like ResNet are first taught on ImageNet (14M distinct photos) to learn what "edges" and "shapes" look like. | Pre-training: By the time the model sees a spine X-ray, it already knows how to see. It only needs to "fine-tune" its knowledge for bones, saving massive amounts of training time. |
| FCN (Fully Convolutional Network) | Segmentation Network | Pixel-wise Classification: FCNs describe every pixel as "bone" or "background," while normal CNNs just say "this image has a spine." It replaces dense layers with convolutional ones, so it can work with images of any size. | It makes a "mask" over the vertebrae, which lets you divide the vertebrae more precisely than with just a bounding box. |
| U-Net | Segmentation Network | Encoder-Decoder with Bridges. It's shaped like a "U." The "where" part of the left side (Encoder) makes the picture smaller to get context. The right side (Decoder) builds it back up to exact information ("what"). Skip connections and keep sharp edges by connecting both sides. | For Medical, even with a few training images, it is great at tracing the exact outlines of organs and bones. |
| 2D-U-Net | Segmentation Network | Slice-by-Slice Processing: Uses the U-Net design on one 2D slice of a CT scan at a time instead of the whole 3D volume. | It needs less computer memory than the 3D versions, so it's easier to run on regular hospital computers. |
| nnU-Net (No-New-Net) | Auto-ML Framework | Self-Adaptation: This is a "smart" U-Net that looks at the dataset (for example, image resolution and spacing) and instantly figures out the best way to set up the network (layer depth and patch size) without anyone having to guess. | It removes human error in design. In the study, it achieved 10x speed because it was perfectly optimized for the specific CT variation used. |
| SNN (Stereo Neural Network) | Depth Estimation | Disparity Matching: In this study, SNN stands for Stereo and not Spiking. It uses two pictures, like left and right "eyes," and figures out depth by looking at how things move between the two views. | It makes it possible to move through 3D space with basic cameras in place of costly CT machines that use radiation during surgery. |

**Table 2**: (continued)

| **Model** | **Type** | **Core Working Principle (The "How")** | **Why does it fit this task?** |
| --- | --- | --- | --- |
| YOLOv8m (You Only Look Once) | Object Detection | Single-Shot Grid: It divides the image into a grid. For every cell, it predicts "Is there an object center here?" and "How big is the box?" simultaneously. It does this in one single pass. | Real-Time Speed: Because it only looks once (unlike older models that look hundreds of times), it is fast enough to track surgical tools or vertebrae in live video. |
| Supervised 3D CycleGAN | Generative Adversarial Network (GAN) | Cycle Consistency: It uses two competing networks: a Generator (creates fake CTs from MRI) and a Discriminator (tries to catch the fakes). It ensures that if you convert MRI >>> CT >>> MRI, you get the original image back. | It allows surgeons to "see" a CT scan (good for bone) derived from an MRI (good for nerves) without exposing the patient to the radiation of an actual CT scan. |
| SafeRPlan | Deep Reinforcement Learning (DRL) | Constrained Agent: An AI "agent" learns by trial and error in a simulated environment. Unlike standard RL, SafeRPlan adds hard "penalties" or constraints preventing it from ever choosing a path that touches vital organs. | It automates the planning of screw trajectories. It doesn't just "see" the spine; it decides the best path to drill. |

Various AI models were applied to automate surgical tasks. These are divided into four groups: Segmentation (outlining bones), Detection (localizing landmarks or implants), Registration (aligning preoperative plans with intraoperative anatomy), and Others (such as image enhancement and modality synthesis), as shown in **Table 1** and **Fig. 2**. The results are described below.

**Segmentation**

Various model types were identified, including FCN, nnU-Net, and 2D-U-Net, alongside unspecified ML and DL architectures. In terms of pure accuracy, results varied by data source. As an example, Burström et al. (5) found an automated segmentation accuracy of 86.1%, which increased up to 95.4% when patients with severe spinal deformities were not evaluated. Similarly, the FCN model by Esfandiari et al. (9) achieved 93.0% accuracy on synthetic X-rays, though this dropped to 83.0% on clinically realistic images.

Pose Estimation and Geometric Accuracy beyond simple segmentation, the spatial accuracy of these models was critical. Esfandiari et al. found a 3D angular discrepancy of 1.93° ± 0.64° between the estimated screw axis and the measured tunnel axis, with a Euclidean distance error of 1.92 ± 0.55 mm. Using nnU-Net, Scherer et al. (13) found a bit higher mean absolute differences (MADs): 4.61 ± 2.27 mm for the point of the screw head and 5.51 ± 3.64° for the direction of the screw. Even though there were some differences, the clinical safety was high. Using the Gertzbein-Robbins system, 3.8% of the screws were Grade B (not completely within boundaries) and 96.2% were Grade A (completely within boundaries), as shown in **Fig. 3A** (23).

Generalization and Validation: newer models tested robustness across different datasets. Da Mutten et al. (21) showed that a 2D-U-Net could generalize well, achieving a Dice score that actually increased from internal validation (0.76 ± 0.12) to external validation (0.79 ± 0.17).

Efficiency Gains: A consistent finding across studies (5, 12, 19, 20) was the reduction in processing time. Most notably, Scherer et al. demonstrated a 10-fold increase in speed, reducing the planning time from 6.41 minutes (manual) to just 41.8 seconds (automated) per case. Burström et al. similarly reported a mean time of 11 ± 4 seconds for 5 vertebrae.

Clinical Workflow: Accurate segmentation is merely the prerequisite for 3D reconstruction and trajectory planning (20, 29). As these studies highlight, while automated segmentation significantly accelerates the preoperative workflow by replacing manual plotting, clinical validation (post-validation) remains essential to ensure these time gains do not come at the cost of geometric precision.

**Landmark and object detection**

High-Precision Landmark Detection: A safe plan for the trajectory depends on correctly identifying the anatomical landmarks. In controlled studies, AI models demonstrated exceptional reliability. At a 3 mm distance threshold, Zhang et al. (14) utilized a modified U-Net and ResNet34 architecture that could automatically find the landmark on the lumbar vertebrae. It had a PCK of more than 93%. Additionally, the intraclass correlation coefficients (ICC) for seven parameters, including pedicle axial angle, screw path length, pedicle width, and interpedicular distance, were 0.82–0.98, supporting the clinician's assessment.

Screw and Implant Detection: AI can detect each pedicle screw implant, and its application is effective for spinal implant identification. Yang et al. reported a comparison of three AI models. They found the ResNet34 model, with ImageNet pre-trained weights and transfer learning, achieved 97.0% accuracy and a 96.7% recall for anteroposterior (AP) radiography, as well as a 98.7% accuracy and a 98.2% recall for lateral (Lat) radiography. Google AutoML demonstrated 91.4% precision and 87.4% recall for AP radiography, whereas Lat radiography showed 97.9% precision and 98.4% recall. In Apple Create ML, AP radiography demonstrated 76.0% precision and 73.0% recall, while Lat radiography revealed 89.0% precision and 87.0% recall. (16)

Safety and Grading Validation: Several studies moved beyond simple detection to clinical safety verification. Siemionow et al. (12) used a CNN to grade screw placement, reporting that 100.0% of 208 samples were classified as Zdichavsky Grade (24) IA (no perforation), as shown in **Fig. 3B**. Similarly, under the Gertzbein-Robbins system, as shown in **Fig. 3A**, 99.0% (206/208) were Grade A.

The Generalization Challenge: Despite these successes, robustness remains a critical issue when models face external data. Da Mutten et al. used the YOLOv8m model for object detection, achieving the average of the mean average precision calculated at varying IoU thresholds, ranging from 0.50 to 0.95 (mAP50-95) values of 0.64, 0.63, and 0.09 for training, internal validation, and external validation, respectively. Then they utilized another model for automatic segmentation. This drastic drop suggests that while AI can "memorize" specific hospital datasets, it may struggle to detect spinal objects in images from different machines or protocols without further training. (21)

Emerging Technologies: Stereo Vision: To eliminate X-ray dependence, von Atzigen et al. (17) used a Stereo Neural Network (SNN) to autonomously locate pedicle screws with an average inaccuracy of 5.43 mm. In addition, SNN can reconstruct the rod's shape in real time for an accurate 3D shape estimate and rod shape evaluation.

**Registration**

Registration and Real-Time Navigation: Computer-assisted surgery's most significant difficulty is the "registration bridge"—fitting preoperative planning to intraoperative anatomy. Liebmann et al. (15) addressed this using a U-Net architecture to assist navigation. The median registration success rate was 100.0%, with a median Target Registration Error (TRE) of 2.7 mm, Trajectory Error (TrEr) of 1.6°, Entry Point Error (EpEr) of 2.3 mm, and Average Distance Difference (ADD) of 2.6 mm.

Speed and Efficiency: Beyond accuracy, Liebmann et al. (15) highlighted the efficiency of AI-driven registration. The median duration for the registration step is 1475 ms, while the pose update step takes 20 ms.

Robotic Safety and Path Planning: Ao et al. (22) demonstrated the preoperative planning system called “SafeRPlan” for robotic spine surgery, which used preoperative and intraoperative registration, combining real-time observation for continuous path planning for PS. They found that it improved safety by 5.0% compared to existing methods for placing pedicle screws, with 99.0% safety rates based on an evaluation of the real ultrasound (US) reconstruction dataset.

Clinical Significance: This study (15, 22) shows that AI can aid virtual planning and physical implementation of the "handshake". Preoperative planning specifies the course, but perfect registration keeps the instrument on it. For robotic and guided spine surgery, AI is improving at hard, real-time spatial alterations. Very low error rates (TRE < 3mm) and high safety margins (99.0%) show this.

**Others**

Image Enhancement and Modality Synthesis: Beyond segmentation and navigation, AI models are increasingly used to overcome the inherent limitations of medical imaging hardware. Thies et al. (11) improved intraoperative imaging by removing metal artifacts with a modified VGG architecture. CT scans often show a "starburst" visible from screws and rods in revision operations. These hid the bone next to them. The model improves body part visibility during challenging revisions. By selecting these things, the user can adjust the C-arm's path online.

Cross-Modality Synthesis (MRI to CT): A more severe approach is to take diagnostic images without radiation. From MRI data, Roberts et al. (18) created "virtual" CT scans using a Supervised 3D CycleGAN. This allows clinicians to use MRI's enhanced soft-tissue contrast and bone detail for surgery planning without radiation from a CT scan.

Accuracy vs. Safety Trade-offs: While the concept of synthetic CTs is promising, current reliability varies significantly by plane. Roberts et al. found that while sagittal plane measurements were accurate (errors <10.0%), axial plane measurements suffered errors of up to 34.0%. Since pedicle screw width and trajectory are primarily determined in the axial plane, this high error rate presents a significant safety barrier. This discrepancy was reflected in the Intraclass Correlation Coefficients (ICC), where synthetic CTs (ICC 0.60–0.92) failed to match the reliability of real CTs (ICC 0.80–0.96), with Intervertebral Disc Height (IVDH) showing the lowest reliability.


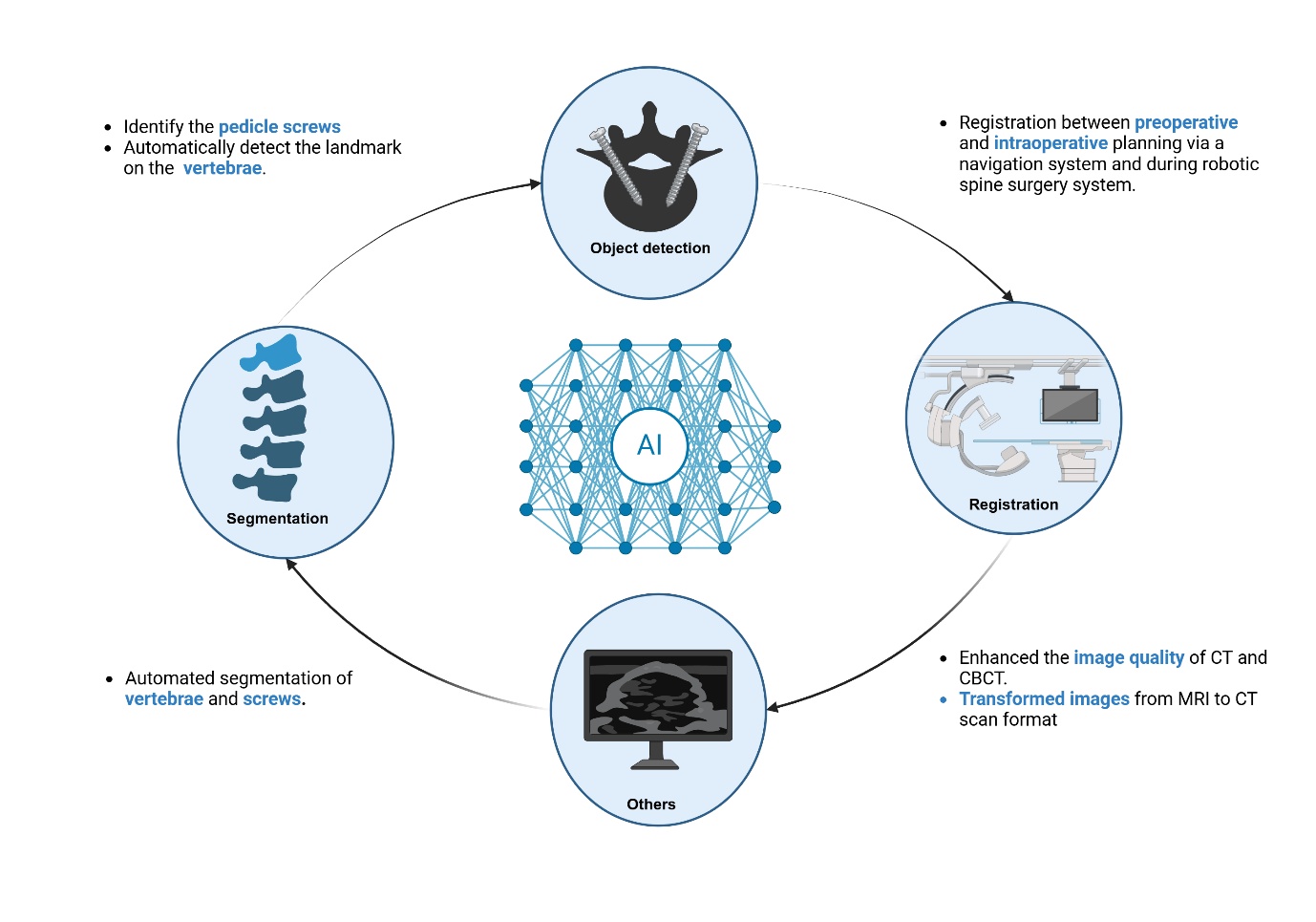


**Figure 2**. The application of AI models related to PS has been divided into four main categories: segmentation, object detection, registration, and others.


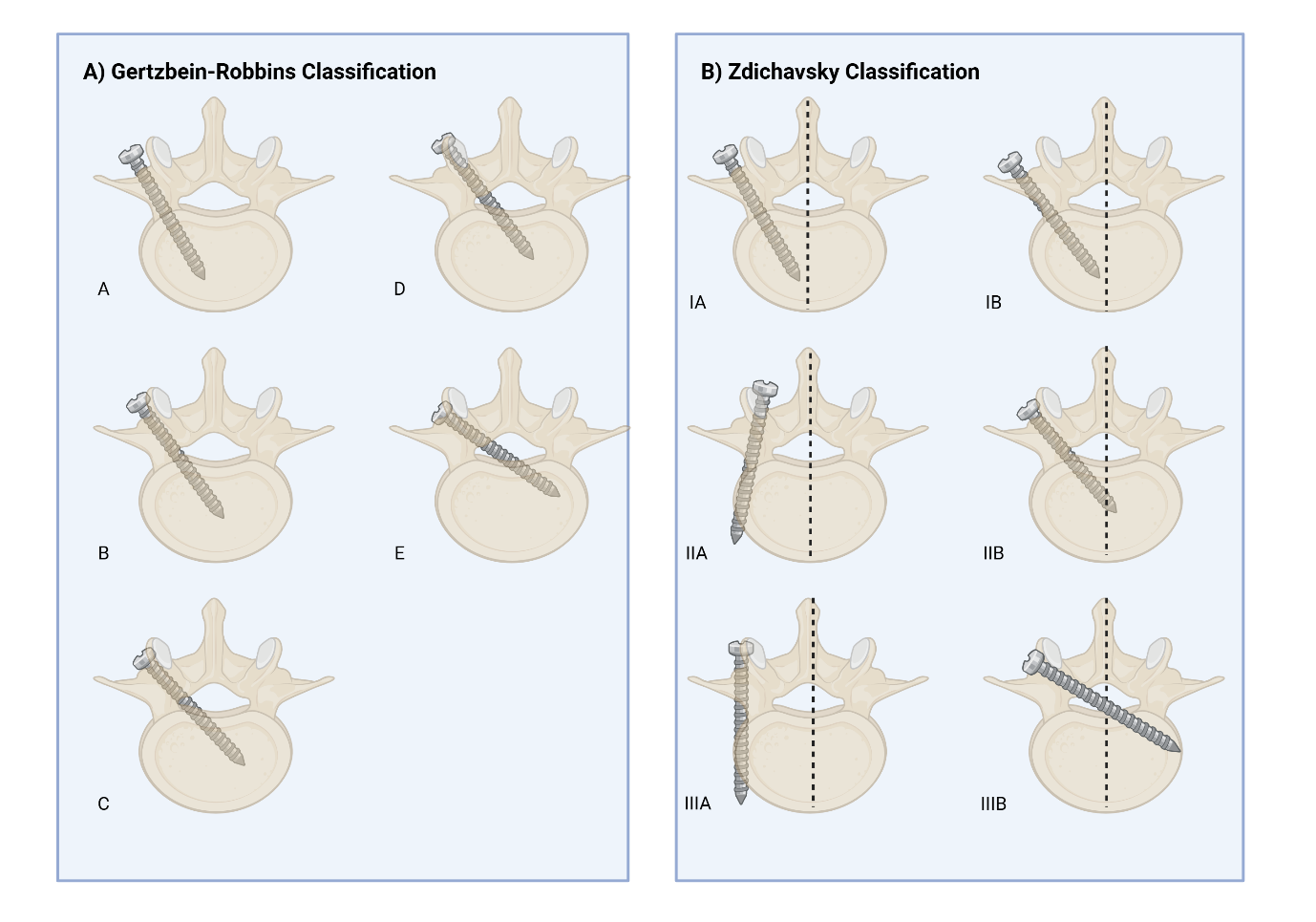


**Figure 3.** Classification systems for assessing pedicle screw placement accuracy. (A) The Gertzbein-Robbins classification system categorizes screw placement based on the degree of cortical breach. Grades A and B are generally considered clinically acceptable. (B) The Zdichavsky classification provides a more detailed assessment of perforation direction and severity. Both systems are widely used in clinical practice and research to evaluate pedicle screw placement safety.

**Figure 3.** Pedicle Screw Placement Accuracy Classification Systems.

Panel A: Gertzbein-Robbins Classification;

Grade A: The screw is fully within the pedicle (without breach),

Grade B: ≤ 2 mm perforation,

Grade C: 2 < x ≤ 4 mm perforation,

Grade D: 4 < x ≤ 6 mm perforation, and

Grade E: > 6 mm perforation.

Panel B: Zdichavsky Classification;

Grade IA: ≥ 50.0% of screw within the pedicle AND ≥ 50.0% of pedicle screw within the vertebral body,

Grade IB:  > 50.0% of pedicle screw lateral outside the pedicle AND > 50.0% of pedicle screw within the vertebral body,

Grade IIA: ≥ 50.0% of pedicle screw within the pedicle AND > 50.0% of pedicle screw lateral outside the vertebral body,

Grade IIB: ≥ 50.0% of pedicle screw within the pedicle and tip of pedicle screw crossing the midline of the vertebral body,

Grade IIIA: >50.0% of pedicle screw lateral outside the pedicle AND >50.0% of pedicle screw lateral outside the vertebral body, and

Grade IIIB: >50.0% of pedicle screw medial outside the pedicle and tip of pedicle screw crossing midline of the vertebral body.

**Risk of Bias Assessment**

The quality assessment of the included studies revealed significant sources of bias, particularly concerning study design, as shown in **Fig. 4**. High-risk **patient selection** was found in 11 (78.6%) of 14 studies. Because the data were retrospective and the researchers used convenience sampling, which may limit the generalizability of the findings. For the **Index Test**, 7 research studies (50.0%) were high risk because they didn't have criteria for evaluating models, which could mean that the performance metrics were too optimistic. In contrast, the **Reference Standard** domain exhibited the least bias because proven clinical grading systems (e.g., Gertzbein-Robbins) were consistently utilized to confirm surgical outcomes, ranking 12 research studies (85.7%) as low risk. Finally, because the duration between the first test and the reference standard confirmation and patient loss was not clearly documented, the risk of bias related to **flow and timing** was generally unknown in 9 studies (64.3%).


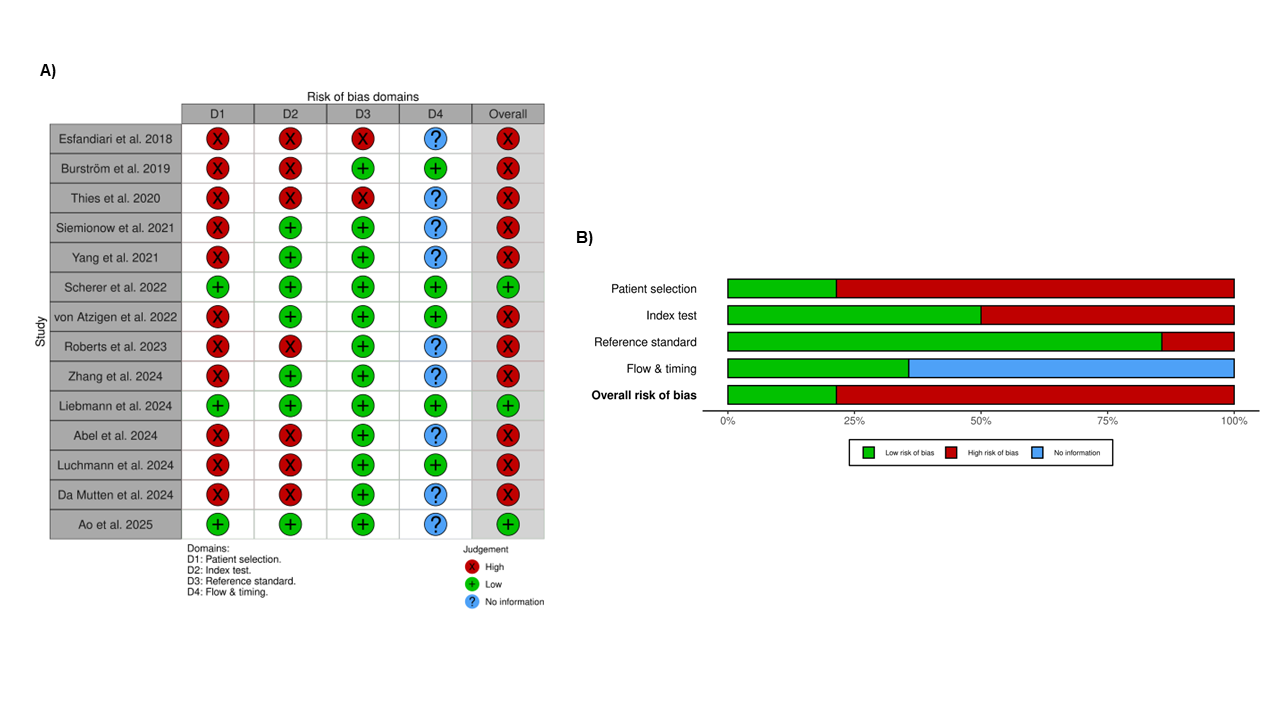


**Figure 4.** Risk of bias assessment using the Robvis risk of bias tool. A) The risk of bias summary displays the review author's judgment regarding each risk of bias item for every included study. B) Risk of bias graph showing the review author's judgment about each risk of bias item presented as percentages across all included studies.

**Performance Metrics reported**

Most of the studies (10 out of 14, or 71.4%) used technical numerical scores, but four studies (28.6%) used only clinical grading. For segmentation tasks, half of the studies reported results, but only three clearly said they used the Dice coefficient. Precision and Recall were also rare; they only showed up in two papers. Most of the people who answered (57.1%) focused on spatial accuracy instead of these usual AI measures. They talked about errors in millimeters or degrees. No study showed AUC/ROC graphs, as shown in **Table S3**.

**Discussion**

**The Evolution of Navigation, From Robotics to AI**: Various AI models were utilized within the perioperative period (pre-, intra-, and postoperative) of PS to enhance surgical outcomes and minimize complications. In general, the medical image analysis methods can be grouped into five categories, including registration, localization, classification, detection, and segmentation. (6) But we classified the AI applications from 14 articles into segmentation (5, 9, 12, 13, 19-21), landmark and object detection (12, 14, 16, 17, 21), registration (12, 16), and other fields, including quality image improvements (11) and image transformation (18). Currently, the AI implementation for PS remains limited, requiring further studies to close these gaps. Traditional freehand PS requires experienced surgeons to ensure screw accuracy and avoid complications. The development of robotic-assisted systems (RAS) applied to preoperative planning (PrO) and intraoperative guidance (IO) is improving surgical accuracy and reducing surgical complications. (25, 26) However, RAS demonstrated limitations, including registration errors, spine movement post-registration, patient body habitus, artifacts from metallic implants, poor bone differentiation, skiving, soft-tissue interference, and physical constraints (26), and took a lot of time. (13) Therefore, different researchers explored solutions to these problems; AI was one approach that was utilized to improve each step of the perioperative period.

**Segmentation, Workflow Efficiency vs. Clinical Generalization**: Medical image segmentation is now emerging as an important research area in the field of computer vision, which uses technology for processing computer images to analyze and process 2D or 3D images to achieve segmentation, extraction, and 3D reconstruction. (27) Several studies used AI models for segmentation to diagnose and treat pathological conditions, such as brain tumor segmentation (28), lung segmentation (29), liver tumor segmentation (30), and others. (27) The accuracy of the segmentation of image modalities such as CT, MRI, and X-rays of spines is also critical for the treatment and diagnosis of pathological diseases of spines, which enhances the PrO phase. (31) While Scherer et al. reported a 10-fold reduction in planning time, the clinical impact depends on whether this time saving translates to reduced operative time or improved patient outcomes—neither of which was measured. This proposed method has the potential to improve workflows in spine surgery when integrated into RAS. (13) Several studies have shown that AI models enhance accuracy, decrease time consumption, and minimize radiation exposure in comparison with traditional methods. (5, 12, 19-21) Moreover, Esfandiari et al. reported the screw segmentation, demonstrating high accuracy using a human X-ray dataset, but the proposed model shows high accuracy only when using the synthetic X-rays; meanwhile, lower accuracy when tested with Realistic X-rays. They also evaluated the angle of the segmented screws in the PO phase compared with the real screws in a porcine specimen analog, which showed high accuracy. Additionally, pose estimation must be evaluated in real clinical settings using real patient X-rays. (9) However, the small number of samples and lack of diversity in the dataset impaired the model's generalization and efficacy. These are the weaknesses that must be addressed in future studies.

**Landmark Detection and the Generalization Challenge**: Landmark and object detection using AI models were applied to a variety of medical image modalities for diagnosis and surgical planning, including colon polyp detection, gallstone detection, cervical cancer detection, mandible fracture detection, stroke lesion detection, pulmonary tuberculosis detection, COVID-19 detection, and others. (32) The automated localization of whole-spine lateral radiographs using DL architecture demonstrated a high degree of accuracy in agreement with manual measurements reported by Yeh et al. (33). In contrast with the PS approach, the AI applied to screw instruments, not anatomical structures or lesions. AI models applied the pedicle screw position, orientation, length, diameter, and screw structures, revealing high detection accuracy. (12, 16, 17) However, the anatomical structure detection, like vertebrae and point landmarks detection for PrO, was reported. (14, 21) According to the articles, researchers showed that AI applications had many benefits. For example, they could cut down on the time needed for PrO image-guided surgery while maintaining a highly accurate measurement for the procedure (12), help clinicians find spinal implants, improve clinical practice, and patient care (16), and more. Furthermore, some limitations of the study were discovered. In some studies, the sample set used only lumbar and sacral spines. This may lead to low diversity and accuracy for the model.

**Bridging the Gap, Registration and Real-Time Navigation**: Image registration allows the direct comparison and integration of data acquired from different sources. In medical image analysis, it enables the integration of information from different temporal points and/or imaging modalities. The example demonstrated a fully automated DL framework for 3D multimodal medical image registration of CT and MRI images of the head. (34) Nonetheless, registration remains an error-prone process that may lead to unsafe planning. (22) Liebmann et al. conducted automated image registration between the PrO and IO using a navigation system guided by augmented reality and employing a U-Net architecture. This method was evaluated on a public dataset, achieving a 100.0% success rate in registration, fast implementation, and absence of radiation exposure. (15) Furthermore, a study conducted by Ao et al. established a safe deep reinforcement learning (DRL) planning approach (SafeRPlan) based on registration concepts, relying on the alignment of PrO to the IO anatomy for robotic spine surgery for PS, resulting in safety rates superior to baseline approaches by over 5.0%. (22) The advantages of AI applications for registration include more precise feature detection, improved intensity matching, especially between different types of images, and faster, more accurate alignment. (35) From the clinical implications for resource-limited settings, x-ray-based AI applications demonstrate the accuracy and precision for the localization of the target.

**Image Enhancement and Modality Synthesis**: AI is helping with medical imaging hardware, localization, and guidance. Implant metal makes "starburst" lines on CT/CBCT scans. These stripes obscure bone and complicate revision procedures. (36, 37) The VGG design was updated by Thies et al. (11) to filter artifacts in real time. Improvements include easier visibility and online C-arm orientation changes without surgical interruption. A more radical application is cross-modality synthesis, aiming to eliminate ionizing radiation. Roberts et al. (18) made "virtual" CT scans from MRI data using a Supervised 3D CycleGAN. This method uses MRI's better soft-tissue contrast and CT's clearer bone clarity without using radiation. Don't forget to be careful. The model had 34.0% axial plane errors, even though the sagittal reconstruction was correct. Synthetic CTs aren't good for high-risk instrumentation because they aren't very accurate when it comes to geometry. The axial image is what mostly determines the width of the pedicle screw.

**Implications for Practice and Research**

**Democratizing Spine Surgery: X-ray-based AI in Resource-Limited Settings**. This study found that using X-ray images instead of costly CT or MRI machines could provide accurate outcomes with the assistance of various types of AI models. In Low- and Middle-Income Countries (LMICs), access to intraoperative CT (O-arm) or MRI navigation is often nonexistent. Our analysis suggests that AI can effectively "upgrade" standard radiography to fill this gap. Esfandiari et al. (9) and Yang et al. (16) demonstrated that standard X-rays, when augmented by AI, could achieve segmentation and implant detection accuracies exceeding 90.0%. Furthermore, Luchmann et al. (20) showed that an X-ray-based deep learning system (X23D) actually reduced breach rates (21.0% vs. 24.0%) and radiation exposure compared to conventional freehand fluoroscopy.

**Barriers to Implementation in Southeast Asia**: It might help democracy, but using it in therapy is hard to do, especially in Southeast Asia. Our study has found four main reasons that prevent more people from using these tools. First, the price is still too high. Software can be scaled up or down, but AI-based robotic systems cost between $200,000 and $500,000, which is too expensive for state hospitals in poor countries. Second, most of these places don't have the AI technology they need, like high-performance computers and data tools that can work together to run these models intraoperatively. Third, rules for businesses are often out of date compared to technology. Thailand hasn't accepted many advanced AI-driven guiding systems. This makes it legally and technically risky for people who start using them first. Finally, a lack of local vendor support makes technical downtime worse because local engineers sometimes don't have the skills to work on AI-robotic systems that only some companies build. These problems show that AI can work in places with few resources, but it usually doesn't have what it needs to work.

**The Economic and Infrastructure Advantage**: AI systems that use X-rays have a number of advantages. For example, they are much cheaper than CT or MRI-based navigation systems (standard X-ray machines vs. intraoperative CT/CBCT systems). They also offer a simpler setup, reduced running costs (no maintenance contracts or costly consumables), and are easier to procure in rural locations where X-rays are the main imaging option. However, our review identified important limitations of X-ray-based AI systems.

**The Accuracy Trade-off and** **Future Directions**: However, this accessibility comes at the cost of geometric precision. A distinct "performance gap" remains: X-ray-based segmentation accuracy (83.0–93.0%) lags behind CT-based systems (95–98.0%). (5, 13) Complex 3D anatomical assessment is difficult using 2D projection X-rays, especially in malformed spines. Image quality reduction in high BMI patients impairs AI effectiveness because X-rays cannot detect neuronal structures, blood vessels, or soft tissue pathology that may compromise surgical planning. Scoliosis, kyphosis, and severe degenerative changes diminish X-ray-based AI accuracy, and full assessment requires two orthogonal views, increasing radiation exposure. **Future Direction**: Focus on hybrid AI models that maximize X-ray accuracy in future research. This will maximize clinical value in low-resource areas. They should also construct inexpensive, portable fluoroscopy devices with AI navigation, confirm that X-ray-based AI systems operate for everyone (even spinal abnormalities), and compare and contrast AI navigation with standard methods in resource-poor areas. The globe could have safe and correct PS placement with these adjustments. This would reduce health-based variations in spine surgery outcomes.

**Quality of Evidence and Risk of Bias**

**Risk of Bias Assessment**: Existing evidence must moderate enthusiasm for these technologies. Our rigorous quality assessment found major biases that would invalidate the results. Through retrospective data and convenience sampling, 11 (78.6%) of 14 studies identified high-risk patient selection. This increases selection bias, suggesting models were trained on "clean" or "ideal" cases rather than sequential, real-world patient groups. In addition, 50.0% of Index Test investigations were high risk because they lacked pre-specified criteria for model performance evaluation, raising concerns that accuracy measures may be optimistic or overfit. The Reference Standard shows that 85.7% of studies had low bias due to the consistent use of proven clinical grading systems (e.g., Gertzbein-Robbins) to confirm surgical outcomes. **Challenges in Standardization:** Dice, AUC, and precision aren't always available, making it challenging to compare AI systems and acquire repeatable results. Since they're the most used instruments, current research focuses on making measures usable in surgery (mm/degrees) and clinical grading (Gertzbein-Robbins) rather than standardizing computations. This feature makes it clinically valuable, but without rigorous scientific reporting, algorithms cannot be objectively compared. Future research needs "dual-reporting" standards. This requires technical measures for validation and clinical grading for usefulness. **Clinical Readiness Assessment:** Of 14 studies, only 5 (35.7%) involved prospective design, and none were randomized controlled trials comparing AI-assisted vs. traditional approaches. Consequently, before routine clinical adoption, the following are essential: (1) prospective multicenter trials, (2) head-to-head comparisons with conventional navigation, (3) long-term patient outcome data, (4) cost-effectiveness analyses, (5) external validation across diverse populations.

**Limitations and Methodological Quality**: The quality of the proof we have must temper our excitement about these technologies. Our methodical study finds three major problems that make it hard to use this in clinics: **Anatomical Bias**: A big problem is that most datasets leave out the thoracic spine. There is a big problem with how widely the results can be applied because only two studies looked at vertebrae in the chest. Because thoracic pedicles are quite a bit smaller and differently shaped compared to lumbar pedicles, AI performance metrics based on lumbar datasets probably rate the accuracy of the thoracic area too highly. **Heterogeneity and Standardization**: Substantial heterogeneity in AI models, datasets, and outcome measures precluded quantitative synthesis. A major limitation of current literature is the paucity of standardized algorithmic performance metrics. Only 64.3% of studies reported objective AI metrics (precision, recall, Dice coefficient), while 35.7% relied solely on clinical grading systems. This prevents meaningful comparison across studies and assessment of whether AI performance improvements translate to clinical benefit. For example, high Gertzbein-Robbins Grade A rates may reflect conservative screw planning rather than AI algorithmic accuracy. However, our structured narrative approach using the PICO framework allows systematic comparison of findings and identification of evidence gaps. **Lack of Pathological Diversity**: The reliance on small datasets from the past means that complicated conditions like severe scoliosis, osteoporosis, or revision cases are often left out. Because there isn't any variety, the model gets overfit, which makes it less robust in difficult real-world situations. **The "Sim-to-Real" Gap**: This is a problem in technology—models that are taught on clean, synthetic data don't work well when they're used on clinical images from the real world that have noise, artifacts, and soft-tissue interference.

**Authors' Experience & Pilot Study:** We're using AI-assisted methods in our clinical workflow to overcome this review's issues, especially the lack of pre-study data. Despite the learning curve, AI-driven pre-planning helps detect complicated defects that 2D fluoroscopy misses. Personal evidence isn't enough. After completion, the IRB will approve our school's pilot project. The study directly compares AI-assisted planning versus manual planning. This prospective study in Thai tertiary care will assess accuracy (breach rates), operation duration, and radiation exposure. We think region-specific statistics are vital to demonstrate that modernizing spine surgery in Southeast Asia is worth the costs and permissions.

**Conclusion**

This systematic review of 14 studies demonstrates that AI applications in lumbar pedicle screw fixation show promise for automated planning, improved accuracy, and reduced operative time, but current evidence remains insufficient for routine clinical adoption. AI-based segmentation reduces planning time tenfold while maintaining accuracy, and detection algorithms achieve >93% landmark identification. However, all studies have significant methodological limitations, including retrospective designs, small sample sizes, limited diversity in datasets, and the absence of prospective comparative trials. Before clinical implementation, rigorous multicenter randomized trials comparing AI-assisted versus conventional techniques are essential, along with validation across diverse patient populations and spinal pathologies. Currently, AI should be viewed as an emerging educational and planning tool rather than a replacement for surgeon expertise and established navigation methods.

**Author contribution:**

1. Conceptualization: Sorayouth Chumnanvej, Pakpoom Thintharua, and Ratchaphon Prabrai
2. Data collection, analysis, writing manuscript, and revising manuscript: Pakpoom Thintharua, Ratchaphon Prabrai, Anuyut Khamsiriwatchara, and Rohan Sethi
3. Analysis, writing manuscript, revising, and finalizing manuscript: Sorayouth Chumnanvej

All authors have read and agreed to the published version of the manuscript.

**Declarations**

**Funding**

Funding: Not applicable.

**Clinical Trial Registration**

Clinical trial number: not applicable.

**Table S1**: Complete search strategy with database-specific syntax

| **Database** | **Search Strings** | **Exclusion** | **Filters** | **Search Date** |
| --- | --- | --- | --- | --- |
| PubMed | ((("Pedicle Screws"[Mesh]) AND ("Artificial Intelligence"[Mesh])) OR (("Pedicle Screws"[Mesh]) AND ("Neural Networks, Computer"[Mesh]))) AND ((("Pedicle Screws"[Mesh]) AND ("Radiography"[Mesh])) OR (("Pedicle Screws"[Mesh]) AND ("Magnetic Resonance Imaging"[Mesh]))) | Review and meta-analysis, letter, Case Reports, Editorial, Books, and Documents | Published 2015-2025, English, Human subjects | January 15, 2025 |
| BASE | Pedicle Screws AND (Artificial Intelligence OR Neural Networks, Computer) AND Pedicle Screws AND (Radiography OR Magnetic Resonance Imaging) | Review and meta-analysis, letter, Case Reports, Editorial, Books, and Documents | Published 2015-2025, English, Human subjects | January 15, 2025 |
| ScienceDirect | (("Pedicle Screws" AND "Artificial Intelligence") OR ("Pedicle Screws" AND "Neural Networks, Computer")) AND (("Pedicle Screws" AND "Radiography") OR ("Pedicle Screws" AND "Magnetic Resonance Imaging")) | Review and meta-analysis, letter, Case Reports, Editorial, Books, and Documents | Published 2015-2025, English, Human subjects | January 16, 2025 |

**Table S2**: Detailed QUADAS-2 assessments

| **Patient Selection** | **Index Test (AI Model)** | **Reference Standard** | **Flow and Timing** | **Overall Risk** |
| --- | --- | --- | --- | --- |
| HIGH RISK: Retrospective design (Phantom/Synthetic data) | HIGH RISK: Evaluated on synthetic X-rays; lack of clinical threshold | HIGH RISK: Experimental measures (tunnel axis) vs validated clinical grading | UNCLEAR RISK: Insufficient reporting of time interval | HIGH |
| HIGH RISK: Convenience sampling (21 Human cadavers) | HIGH RISK: Unidentified ML model properties | LOW RISK: Clinical accuracy (verified manually) | LOW RISK: Mean time 11 ± 4 sec reported | HIGH |
| HIGH RISK: Retrospective (Public TCIA dataset) | HIGH RISK: Subjective visual quality assessment | HIGH RISK: Visual assessment (No formal grading system) | UNCLEAR RISK: Insufficient reporting | HIGH |
| HIGH RISK: Retrospective (20 patients, convenience) | LOW RISK: Validated autonomous planner | LOW RISK: Validated grading (Zdichavsky, Gertzbein, Ravi) | UNCLEAR RISK: Insufficient reporting | HIGH |
| HIGH RISK: Retrospective (2894 lumbar spine segments) | LOW RISK: ResNet34/AutoML with clear metrics | LOW RISK: Radiographic ground truth labels | UNCLEAR RISK: Insufficient reporting | HIGH |
| LOW RISK: Consecutive registry data (179 patients) | LOW RISK: nnU-Net (Standardized architecture) | LOW RISK: Validated grading (Gertzbein-Robbins) | LOW RISK: "10 times superior speed" reported | LOW |
| HIGH RISK: Convenience sampling (8 Human cadavers) | LOW RISK: SNN with clear error metrics | LOW RISK: Standard error measurement (mm) | LOW RISK: Time reported (231s vs 476s) | HIGH |
| HIGH RISK: Retrospective (20 patients, <30 days) | HIGH RISK: CycleGAN (Generative model validation issues) | LOW RISK: Real CT measurements (Gold standard) | UNCLEAR RISK: Insufficient reporting | HIGH |
| HIGH RISK: Retrospective (282 patients) | LOW RISK: Pre-defined landmarks and thresholds | LOW RISK: Clinician evaluation & ICC | UNCLEAR RISK: Insufficient reporting | HIGH |
| LOW RISK: Prospective design (SpineDepth validation) | LOW RISK: U-Net with clear metrics | LOW RISK: Standard metrics (TRE, TrEr) | LOW RISK: Registration time 1475 ms | LOW |
| HIGH RISK: Retrospective (48 consecutive exams) | HIGH RISK: Unidentified DL model | LOW RISK: CT scan geometry (3D reconstruction) | UNCLEAR RISK: Insufficient reporting | HIGH |
| HIGH RISK: Convenience sampling (Ex-vivo/Cadaver) | HIGH RISK: Unidentified DL model | LOW RISK: Breach rate/Radiation metrics | LOW RISK: Time reported (167s vs 156s) | HIGH |
| HIGH RISK: Retrospective (Public datasets: VerSe, MSD) | HIGH RISK: Training/Val split only (No external threshold) | LOW RISK: Standard Dice/mAP scores | UNCLEAR RISK: Insufficient reporting | HIGH |
| LOW RISK: Prospective (SafeRPlan validation) | LOW RISK: Deep RL with safety evaluation | LOW RISK: Safety rates on real US reconstruction | UNCLEAR RISK: Time not explicitly reported in summary | LOW |

**Table S3**: AI Performance Metrics Summary

| **Authors** | **AI Application** | **Algorithmic Metrics Reported** | **Clinical / Functional Metrics** | **Metric Assessment** |
| --- | --- | --- | --- | --- |
| Burström et al. (2019) | Segmentation | Segmentation Accuracy | Algorithmic Acc: 86.1%  Clinical Acc: 95.4%  Time: 11 ± 4 s | Missing standard segmentation metrics (Dice, IoU); relies on general accuracy. |
| Esfandiari et al. (2018) | Segmentation, Pose Estimation | Segmentation Accuracy, Geometric Error | Accuracy: 93.00% (synthetic), 83.00% (clinical)  Ang. Error: 1.93° ± 0.64°  Tip Error: 1.92 ± 0.55 mm | Missing Dice/IoU; relies on "Accuracy" and geometric error. |
| Thies et al. (2020) | Image Quality Improvement | None | Qualitative improvement only | Qualitative only. Missing all quantitative metrics (PSNR, SSIM, etc.). |
| Siemionow et al. (2021) | Segmentation, Landmark Detection | None (Clinical Grading only) | Grading: 100% Zdichavsky Grade IA; 99% Gertzbein Grade A | Clinical grading only. Missing algorithmic performance data. |
| Scherer et al. (2022) | Segmentation | Dice, MAD | Mean Dice: 0.61 ± 0.16  MAD: 3.96 mm (tip), 5.51° (dir)  Grading: 96.2% Gertzbein Grade A | Complete. Reports standard AI metric (Dice) alongside clinical error/grading. |
| Zhang et al. (2024) | Landmark Detection | PCK, ICC | PCK: >93% (at 3mm threshold)  ICC: 0.82–0.98 (various parameters) | Good use of detection-specific metric (PCK) and reliability (ICC). |
| Liebmann et al. (2024) | Registration, Segmentation | DSC, TRE, Success Rate | DSC: 0.74  Success Rate: 100%  TRE: 2.7 mm | Complete. Comprehensive reporting of registration error (TRE) and segmentation (DSC). |
| Yang et al. (2021) | Object Detection | Precision, Recall, Accuracy | ResNet34: Acc 97.0–98.7%; Recall 96.7–98.2%  AutoML: Precision 91.4–97.9%; Recall 87.4–98.4%  Create ML: Precision 76.0–89.0%; Recall 73.0–87.0% | Complete. Detailed breakdown of standard classification metrics. |
| von Atzigen et al. (2022) | Object Detection | Detection Success Rate | Avg Error: 5.43 mm  Time: Reduced (231s vs 476s) | Missing standard detection metrics (mAP, Precision); focuses on surgical efficiency. |
| Roberts et al. (2023) | Image Synthesis (MRI to CT) | Geometric Error, ICC | Error: <10% (Sagittal), up to 34% (Axial) (Uses MAE for translation accuracy)  ICC: 0.60–0.92 (Synthetic vs Real) | Missing synthesis metrics (SSIM, PSNR); relies on ICC and geometric measurement. |
| Abel et al. (2024) | Segmentation | ICC | ICC 0.62–0.92 (MRI vs CT agreement) | Reports reliability (ICC) but missing standard segmentation overlap metrics (Dice). |
| Luchmann et al. (2024) | Segmentation | None (Clinical Grading only) | Breach Rate: 21% (implies grading was performed)  Radiation: 33.26 mGy | Clinical grading only. Compares surgical outcomes rather than algorithm performance. |
| Da Mutten et al. (2024) | Object Detection, Segmentation | mAP, Dice | mAP50-95: 0.09–0.64  Mean Dice Score: 0.75–0.79 (Internal Validation) | Complete. Reports standard technical metrics for both detection and segmentation. |
| Ao et al. (2025) | Registration | Safety Rate | 99% (defined by breach < 2mm, i.e., Gertzbein A)  Safety Improvement: 5% | Missing RL-specific convergence or registration error metrics; relies on "Safety". |

Acc: Accuracy, ADD: Average Distance Difference, Avg: Average, DSC: Dice Similarity Coefficient, EpEr: Entry Point Error, ICC: Intraclass Correlation Coefficient, IoU: Intersection over Union, MAD: Mean Absolute Difference, mAP: Mean Average Precision, MRI: Magnetic Resonance Imaging, PCK: Percentage of Correct Keypoints, PSNR: Peak Signal-to-Noise Ratio (not reported, listed as missing), RL: Reinforcement Learning, SSIM: Structural Similarity Index Measure (not reported, listed as missing), TRE: Target Registration Error, TrEr: Trajectory Error.

**Table S4**: PRISMA 2020 checklist

| **Section and Topic** | **Item #** | **Checklist item** | **Location where item is reported** |
| --- | --- | --- | --- |
| **TITLE** | | |  |
| Title | 1 | Identify the report as a systematic review. |  |
| **ABSTRACT** | | |  |
| Abstract | 2 | See the PRISMA 2020 for Abstracts checklist. |  |
| **INTRODUCTION** | | |  |
| Rationale | 3 | Describe the rationale for the review in the context of existing knowledge. |  |
| Objectives | 4 | Provide an explicit statement of the objective(s) or question(s) the review addresses. |  |
| **METHODS** | | |  |
| Eligibility criteria | 5 | Specify the inclusion and exclusion criteria for the review and how studies were grouped for the syntheses. |  |
| Information sources | 6 | Specify all databases, registers, websites, organisations, reference lists and other sources searched or consulted to identify studies. Specify the date when each source was last searched or consulted. |  |
| Search strategy | 7 | Present the full search strategies for all databases, registers and websites, including any filters and limits used. |  |
| Selection process | 8 | Specify the methods used to decide whether a study met the inclusion criteria of the review, including how many reviewers screened each record and each report retrieved, whether they worked independently, and if applicable, details of automation tools used in the process. |  |
| Data collection process | 9 | Specify the methods used to collect data from reports, including how many reviewers collected data from each report, whether they worked independently, any processes for obtaining or confirming data from study investigators, and if applicable, details of automation tools used in the process. |  |
| Data items | 10a | List and define all outcomes for which data were sought. Specify whether all results that were compatible with each outcome domain in each study were sought (e.g. for all measures, time points, analyses), and if not, the methods used to decide which results to collect. |  |
|  | 10b | List and define all other variables for which data were sought (e.g. participant and intervention characteristics, funding sources). Describe any assumptions made about any missing or unclear information. |  |
| Study risk of bias assessment | 11 | Specify the methods used to assess risk of bias in the included studies, including details of the tool(s) used, how many reviewers assessed each study and whether they worked independently, and if applicable, details of automation tools used in the process. |  |
| Effect measures | 12 | Specify for each outcome the effect measure(s) (e.g. risk ratio, mean difference) used in the synthesis or presentation of results. |  |
| Synthesis methods | 13a | Describe the processes used to decide which studies were eligible for each synthesis (e.g. tabulating the study intervention characteristics and comparing against the planned groups for each synthesis (item #5)). |  |
|  | 13b | Describe any methods required to prepare the data for presentation or synthesis, such as handling of missing summary statistics, or data conversions. |  |
|  | 13c | Describe any methods used to tabulate or visually display results of individual studies and syntheses. |  |
|  | 13d | Describe any methods used to synthesize results and provide a rationale for the choice(s). If meta-analysis was performed, describe the model(s), method(s) to identify the presence and extent of statistical heterogeneity, and software package(s) used. |  |
|  | 13e | Describe any methods used to explore possible causes of heterogeneity among study results (e.g. subgroup analysis, meta-regression). |  |
|  | 13f | Describe any sensitivity analyses conducted to assess robustness of the synthesized results. |  |
| Reporting bias assessment | 14 | Describe any methods used to assess risk of bias due to missing results in a synthesis (arising from reporting biases). |  |
| Certainty assessment | 15 | Describe any methods used to assess certainty (or confidence) in the body of evidence for an outcome. |  |
| **RESULTS** | | |  |
| Study selection | 16a | Describe the results of the search and selection process, from the number of records identified in the search to the number of studies included in the review, ideally using a flow diagram. |  |
|  | 16b | Cite studies that might appear to meet the inclusion criteria, but which were excluded, and explain why they were excluded. |  |
| Study characteristics | 17 | Cite each included study and present its characteristics. |  |
| Risk of bias in studies | 18 | Present assessments of risk of bias for each included study. |  |
| Results of individual studies | 19 | For all outcomes, present, for each study: (a) summary statistics for each group (where appropriate) and (b) an effect estimate and its precision (e.g. confidence/credible interval), ideally using structured tables or plots. |  |
| Results of syntheses | 20a | For each synthesis, briefly summarise the characteristics and risk of bias among contributing studies. |  |
|  | 20b | Present results of all statistical syntheses conducted. If meta-analysis was done, present for each the summary estimate and its precision (e.g. confidence/credible interval) and measures of statistical heterogeneity. If comparing groups, describe the direction of the effect. |  |
|  | 20c | Present results of all investigations of possible causes of heterogeneity among study results. |  |
|  | 20d | Present results of all sensitivity analyses conducted to assess the robustness of the synthesized results. |  |
| Reporting biases | 21 | Present assessments of risk of bias due to missing results (arising from reporting biases) for each synthesis assessed. |  |
| Certainty of evidence | 22 | Present assessments of certainty (or confidence) in the body of evidence for each outcome assessed. |  |
| **DISCUSSION** | | |  |
| Discussion | 23a | Provide a general interpretation of the results in the context of other evidence. |  |
|  | 23b | Discuss any limitations of the evidence included in the review. |  |
|  | 23c | Discuss any limitations of the review processes used. |  |
|  | 23d | Discuss implications of the results for practice, policy, and future research. |  |
| **OTHER INFORMATION** | | |  |
| Registration and protocol | 24a | Provide registration information for the review, including register name and registration number, or state that the review was not registered. |  |
|  | 24b | Indicate where the review protocol can be accessed, or state that a protocol was not prepared. |  |
|  | 24c | Describe and explain any amendments to information provided at registration or in the protocol. |  |
| Support | 25 | Describe sources of financial or non-financial support for the review, and the role of the funders or sponsors in the review. |  |
| Competing interests | 26 | Declare any competing interests of review authors. |  |
| Availability of data, code and other materials | 27 | Report which of the following are publicly available and where they can be found: template data collection forms; data extracted from included studies; data used for all analyses; analytic code; any other materials used in the review. |  |

*From:*  Page MJ, McKenzie JE, Bossuyt PM, Boutron I, Hoffmann TC, Mulrow CD, et al. The PRISMA 2020 statement: an updated guideline for reporting systematic reviews. BMJ 2021;372:n71. doi: 10.1136/bmj.n71. This work is licensed under CC BY 4.0. To view a copy of this license, visit <https://creativecommons.org/licenses/by/4.0/>

**Table S5**: Detailed exclusion reasons

| **Stage** | **Category** | **Specific Reason** | **Count** |
| --- | --- | --- | --- |
| Identification | Total Identified | Initial search (BASE: 1296, PubMed: 53, SciDirect: 143) | 1,492 |
| Deduplication | Duplicate Removal | Duplicates removed (Calculated gap: 1492 - 1168) | -324 |
| Screening | Screened Pool | Articles remaining for Title/Abstract Screening | 1,168 |
|  | Excluded (Screening) | Excluded based on Title/Abstract:  • Review articles, Meta-analyses, Case reports, Letters, Editorials  • Only abstracts / Paid articles (unavailable)  • Irrelevant topic (No relationship between AI and Pedicle Screws) | -1,077 |
| Eligibility | Assessed for Eligibility | Full-text articles assessed | 91 |
|  | Excluded (Eligibility) | Excluded after full-text review:  • Non-English language  • Wrong spine segment (Non-lumbar/lumbosacral)  • Animal studies (Non-Human/Non-Cadaver)  • Outside publication date (2015–2025)  • Insufficient data on the AI model or accuracy | -77 |
| Included | Final Analysis | Studies included in the qualitative synthesis | 14 |

**Table S6**: Comprehensive data extraction

| **Title** | **Authors** | **Publication Year** | **Search engine** | **Study Design** | **Population** | **Sample size, n** | **Perioperative phase** | **Image modalities** | **Applications** | **AI models** | **Data extraction notes (Reader 1)** | **Data extraction notes (Reader 2)** | **Data extraction notes (Agreement)** |
| --- | --- | --- | --- | --- | --- | --- | --- | --- | --- | --- | --- | --- | --- |
| A deep learning framework for segmentation and pose estimation of pedicle screw implants based on C-arm fluoroscopy | Esfandiari, Hooman, et al. | 2018 | Pubmed | Retrospective study | Patients undergoing pedicle screw fixation | 40 patients | Postoperative | X-rays | Segmentation of screws and pose estimation | Fully convolutional networks (FCN) | The accuracy of automated segmentation by AI was 93% for synthetic X-rays and 83% for clinically realistic X-rays. The pose estimation accuracy of this method was demonstrated to be 1.93° ± 0.64° and 1.92 ± 0.55 mm on clinically realistic X-rays. A system can help to provide an intraoperative pedicle screw insertion assessment protocol with minimal interference with a surgical procedure. | For the Screw Segmentation, the proposed model show 93% accuracy on synthetic data and 83% on real clinical images. For pose estimation, the proposed model demonstrated that the angular discrepancy in 3D between the estimated screw axis and the measured tunnel axis is about 1.93° ± 0.64°and the Euclidean distance between the estimated screw tip and the tunnel endpoint averaged 1.92 mm, with a standard deviation of 0.55 mm | In synthetic X-rays and clinically realistic X-rays, the automated segmentation accuracy was 93.00% and 83.00%, respectively. The proposed model for pose estimation showed a 3D angular discrepancy of 1.93° ± 0.64° between the estimated screw axis and the measured tunnel axis. The Euclidean distance between the estimated screw tip and the tunnel endpoint averaged 1.92± 0.55 mm. |
| Machine learning for automated 3-dimensional segmentation of the spine and suggested placement of pedicle screws based on intraoperative cone-beam computer tomography | Burström, Gustav, et al. | 2019 | Pubmed | Prospective Study | Human cadavers | 21 cadavers | Intraoperative | Cone Beam Computed Tomography (CBCT) | Segmentation of spines | Machine learning (unspecific) | The clinically relevant pedicle identification and automatic pedicle screw planning accuracy was 86.1%. By excluding patients with severe spinal deformities and previous surgeries, a clinical accuracy of 95.4% was achieved. The mean time (± SD) for automatic segmentation and screw planning in 5 vertebrae was 11 ± 4 seconds. This has the potential to reduce radiation to the patient as well as the burden on the health care system. | The proposed model demonstrated the automatic vertebral segmentation and pedicle screw planning with an accuracy of about 86.1%. By excluding patients with severe spinal deformities and previous surgeries, a clinical accuracy of 95.4% was achieved. and It tool an average of 11 ± 4 seconds for 5 adjacent vertebrae | An AI model demonstrated automatic vertebral segmentation and pedicle screw planning with an accuracy of about 86.1%. By excluding patients with severe spinal deformities and previous surgeries, a clinical accuracy of 95.4% was achieved. The mean time for automatic segmentation and screw planning in 5 vertebrae was 11 ± 4 seconds. |
| A learning-based method for online adjustment of C-arm Cone-beam CT source trajectories for artifact avoidance | Thies, Mareike, et al. | 2020 | Pubmed, BASE | Retrospective study | Chest CT scans from the Cancer Imaging Archive (TCIA), an open-source dataset for AI training | n/a | Intraoperative | Cone Beam Computed Tomography (CBCT) and Computed Tomography (CT) | Improved quality of the image | Modified VGG architecture | Modified VGG architecture improved image quality, particularly in terms of metal artifacts, adjusting the C-arm trajectory online without interruption. This innovative method allows for the real-time adjustment of the C-arm Cone-beam CT (CBCT) source trajectories, aiming to reduce imaging artifacts. | Tomographic reconstructions of the resulting scene-specific CBCT acquisitions exhibit improved image quality, particularly in terms of metal artifacts | Modified VGG architecture improved image quality, particularly in terms of metal artifacts, adjusting the C-arm trajectory online without interruption. |
| Autonomous lumbar spine pedicle screw planning using machine learning: a validation study | Siemionow, Kris B., et al. | 2021 | BASE | Retrospective study | Patients (unspecific) | 20 patients | Preoperative and intraoperative | Computed Tomography (CT) | Segmentation of spines and landmark (object) detection | The autonomous pedicle screw planner (based on machine learning) and convolutional neural networks (CNNs) | Of the 208 placed pedicle screws, 208 (100%) had a Zdichavsky Score 1A, 206 (99.0%) of all screws were Ravi Grade 1, and Gertzbein Grade A, indicating no breach. The final two screws (1.0%) had a Ravi score of 2 (<2 mm breech) and a Gertzbein grade of B (<2 mm breech). These validated the accuracy of the neural network for pedicle screw placement on the CT image. The neural network can minimize preoperative time while maintaining a high accuracy. | For the Zdichavsky score, overall, 208 samples are in grade IA(100%). For the Gertzbein grading, 206 samples are in Grade A (No breach), only 2 in Grade B(< 2 mm), the same as the Ravi grading system. Limitation of the study: investigated only with lumbar and sacral spines. | For the Zdichavsky score, overall, 208 samples were in grade IA (100%). For Gertzbein grading, 206 samples were in grade A (no breach), and only 2 were in grade B (< 2 mm), the same as the Ravi grading system. Limitation of the study: investigated only with lumbar and sacral spines. |
| Deep Learning Application in Spinal Implant Identification | Yang, Hee-Seok, et al. | 2021 | BASE | Retrospective study | The patients who had lumbar spine one-segment instrument surgery | 2894 lumbar spine | Postoperative | X-rays | Object detection of pedicle screw | The ResNet34 model with ImageNet pretrained weights, Google AutoML, Apple Create ML | AI can identify each pedicle screw implant with 76.0% to 98.7% precision and 72.0% to 98.4% recall. Its application is effective for spinal implant identification. | Standard Deep Neural Network with transfer learning showed 97.0% precision, 96.7% recall on AP radiography, and 98.7% precision with 98.2% recall on Lateral radiography. Google AutoML, AP radiography showed 91.4% precision and 87.4% recall; Lat radiography showed 97.9% precision and 98.4% recall. Finally In Apple Create ML, AP radiography showed 76.0% precision and 73.0% recall; Lat radiography showed 89.0% precision and 87.0% recall | The ResNet34 model, with ImageNet pre-trained weights and transfer learning, achieved a 97.0% accuracy and a 96.7% recall for AP radiography, as well as a 98.7% accuracy and a 98.2% recall for lateral radiography. Google AutoML demonstrated 91.4% precision and 87.4% recall for anteroposterior (AP) radiography, whereas lateral (Lat) radiography showed 97.9% precision and 98.4% recall. In Apple Create ML, AP radiography demonstrated 76.0% precision and 73.0% recall, while Lat radiography revealed 89.0% precision and 87.0% recall. |
| Development and validation of an automated planning tool for navigated lumbosacral pedicle screws using a convolutional neural network | Scherer, Moritz, et al. | 2022 | SciDirect | Retrospective study | Patients was derived from a consecutive registry of navigated spinal instrumentations | 179 patients | Intraoperative | Computed Tomography (CT) | Segmentation and generated screws | nnU-Net | AI was successful in all 130 targeted screws for automated screw planning. The minimal absolute difference (MAD) of the screw was 4.61± 2.27 mm for the screw head, 3.96±2.19 mm for tip points, and 5.51±3.64° for screw direction. Mean Dice coefficient was 0.61±0.16. Automatically planned screws were Gertzbein-Robbins classification [GR] grade A in 96.2% in qualitative validation. Planning time was significantly shorter with the automatic approach (0:41 min vs. 6:41 min). This validation found that automated screw planning is similar to manual screw planning and offers enough accuracy to speed up the surgical process. These results offer a high potential to improve workflows in spine surgery when integrated into navigation or robotic assistance systems. | Comparison of automated screws to manually planed screws showed Mean Absolute Differences (MADs) of screw head point 4.61±2.27 mm, screw tip point 3.96±2.19 mm, screw direction 5.51±3.64°, and Mean Dice Coefficient at 0.61±0.16. The automated planting showed 10 times superior in speed (41.8 seconds per case VS. 6.41 min). A total of 96.2% of screws were classified as Gertzbein-Robbins Grade A (completely within cortical boundaries), and 3.8%(5 crews) were Grade B. The proposed model demonstrates noninferiority to manual planning and provides sufficient accuracy and speed. | A comparison of automated screws and manually planed screws demonstrated mean absolute differences (MADs) of 4.61±2.27 mm for screw head point, 3.96±2.19 mm for screw tip point, 5.51±3.64° for screw direction, and a mean Dice coefficient of 0.61±0.16. The automated planting showed 10 times superior speed (41.8 seconds per case vs. 6.41 min). A total of 96.2% of screws were classified as Gertzbein-Robbins Grade A (completely within cortical boundaries), and 3.8% (5 screws) were Grade B. |
| Marker-free surgical navigation of rod bending using a stereo neural network and augmented reality in spinal fusion | von Atzigen, Marco, et al. | 2022 | BASE | Prospective Study | Human cadavers | 8 cadavers | Intraoperative | Stereo camera images | Object detection | Stereo neural network (SNN) | A marker-free approach can track rod bending in spinal fusion surgeries. AI can reconstruct the shape of the rod in real time, providing a high-precision 3D shape estimation and high accuracy in rod shape estimation. The augmented reality (AR) can be integrated for 3D views of the rod in the surgical field. The study introduces a marker-free approach for tracking rod bending in spinal fusion surgeries, eliminating the need for external fiducial markers. The finding has the potential to support the surgeon in pedicle screw planning when using surgical navigation. | The study demonstrated that the marker-free AR navigation system improved efficiency in spinal fusion surgery by reducing bending time (231s vs. 476s in marker-based AR and 348s in freehand bending) and minimizing rebending maneuvers (0.6 vs. 1.1 and 3.5, respectively). The stereo neural network accurately detected pedicle screw positions with an average error of 5.43 mm, enabling precise rod shaping without external tracking markers. | The study indicated that the marker-free AR navigation system made spinal fusion surgery more efficient by cutting down on the time it took to bend (231s vs. 476s for marker-based AR and 348s for freehand bending) and the number of times it had to be bent again (0.6 vs. 1.1 and 3.5, respectively). With an average error of 5.43 mm, the SNN model correctly identified the locations of the pedicle screws. This allowed for precise rod shaping without the need for external tracking markers. |
| Imaging evaluation of a proposed 3D generative model for MRI to CT translation in the lumbar spine | Roberts, Makenze, et al. | 2023 | SciDirect | Retrospective study | Patients who had CT and MRI scans performed within a 30-day period | 20 patients | Preoperative | Magnetic resonance imaging (MRI) | Convert the type of image (MRI to CT) | Supervised a 3D cycleGan model | AI can generate the CT image from MRI with well-preserved anatomical structures, known as synthesis CT. Overall, inter-rater agreement was good, with the ICC of the real CT images ranging from 0.80 to 0.96 and the ICC of the synthetic CT images ranging from 0.60 to 0.92 | Measurements in the sagittal plane were accurate with relative error under 10%, mostly within 5%, but in the axial plane measurement showed inaccuracies with relative error up to 34%. Real CT ICC ranged from 0.80-0.96, Synthetic CT ICC ranged from 0.60-0.92, with the weakest for intervertebral disc height (IVDH).limitation: lack of axial MRI, Patient positioning, and degenerative change | The synthesis CT images showed that the sagittal plane measurements were accurate, with most of the errors being less than 10%. However, the axial plane measurements were not accurate, with errors of up to 34%. The real CT ICC ranged from 0.80 to 0.96, and the synthetic CT ICC ranged from 0.60 to 0.92. The intervertebral disc height (IVDH) measurement had the lowest ICC. |
| Automated measurement of lumbar pedicle screw parameters using deep learning algorithm on preoperative CT scans | Zhang, Qian, et al. | 2024 | SciDirect | Retrospective study | Patients undergoing pedicle screw fixation | 282 patients | Preoperative | Computed Tomography (CT) | Annotation of point landmarks | Modified U-Net (combined with ResNet34) or VGG16 (limited data availability) architectures, U-Net using ResNet34 as the encoder part and conventional U-net architecture as the decoder part | An AI model found landmarks for screw placement with a Percentage of Correct Key Points (PCK) value of 93.5% to 99.0%. It also agreed with radiologists and a spinal surgeon very well, with intraclass correlation coefficients (ICC) ranging from 0.82 to 0.98. These can offer efficient and precise measurements for clinical applications. | The model successfully identified the landmark automatically, achieving a PCK exceeding 93% at a 3 mm distance threshold. Intraclass Correlation Coefficients (ICCs) ranging from 0.82 to 0.98 for parameters like pedicle axial angle, screw path length, pedicle width, and interpedicular distance. The performance of the developed automatic measurement model was comparable to the reference standards obtained from clinicians' measurements. | The model successfully identified the landmark automatically, achieving a PCK exceeding 93% at a 3 mm distance threshold. The intraclass correlation coefficients (ICC) for parameters like pedicle axial angle, screw path length, pedicle width, and interpedicular distance were all between 0.82 and 0.98, with agreement based on evaluation by the clinician. |
| Automatic registration with continuous pose updates for marker-less surgical navigation in spine surgery | Liebmann, Florentin, et al. | 2024 | BASE | Prospective Study | SpineDepth dataset (mockup spine surgeries performed on ten cadaveric human specimens) The dataset includes RGB-D recordings of where the pedicle screws were placed and individually tracked ground truth poses and shapes of spine levels L1–L5. | 10 cadavers | Intraoperative | RGB or RGBD video sources | Registration and navigation | U-Net architecture | A public dataset shows a median of 100% successful registrations, a median target registration error of 2.7 mm, a median screw trajectory error of 1.6°, and a median screw entry point error of 2.3 mm. The procedure was validated in ex vivo surgery, yielding a 100% screw accuracy and a median target registration error of 1.0 mm. These findings have the potential to fully automate registration approaches using RGB-D data and combine them with augmented reality guidance. | Median registration success rate of 100%, Median Target Registration Error (TRE) 2.7 mm, Median Trajectory Error (TrEr) 1.6°, median Entry Point Error (EpEr) 2.3mm, Median Average Distance Difference (ADD) 2.6 mm., Segmentation Dice Similarity Coefficient (DSC) 0.74 and Median Geodesic Angle Error (MGAE) 14°, Median registration step took 1475 ms and the pose update step took 20 ms. | The median registration success rate is 100%, with a median Target Registration Error (TRE) of 2.7 mm, a median Trajectory Error (TrEr) of 1.6°, a median Entry Point Error (EpEr) of 2.3 mm, and a median Average Distance Difference (ADD) of 2.6 mm. The Segmentation Dice Similarity Coefficient (DSC) is 0.74, and the median Geodesic Angle Error (MGAE) is 14°. The median duration for the registration step is 1475 ms, while the pose update step takes 20 ms. |
| Deep‑learning reconstructed lumbar spine 3D MRI for surgical planning: pedicle screw placement and geometric measurements compared to CT | Abel, Frederik, et al. | 2024 | Pubmed | Retrospective study | Adult patients who planned for initial lumbar spine fusion surgery. | 16 patients | Preoperative | Computed Tomography (CT) and Magnetic Resonance Imaging (MRI) | Segmentation and reconstruction of spines | Deep learning (unspecific) | AI can improve the 3D reconstruction of MRI, which gives anatomical details like CT scans for planning lumbar spine surgery. It gives most geometric measurements, except vertebral body length at L1, L2, and L4, with high reliability in patients referred for lumbar spine surgery. | 3D reconstruction MRI matched CT in most metrics but showed discrepancies in Vertebral body length at L1, L2, and L4. Advantages include reduced radiation exposure and simultaneous visualization of bone and soft tissues. | AI can improve the 3D reconstruction of MRI, which gives anatomical details like CT scans for planning lumbar spine surgery. It gives most geometric measurements, except vertebral body length at L1, L2, and L4, with high reliability in patients referred for lumbar spine surgery. |
| Spinal navigation with AI-driven 3D-reconstruction of fluoroscopy images: an ex-vivo feasibility study | Luchmann, Dietmar, et al. | 2024 | Pubmed, BASE | Prospective Study | Human cadavers (torso) with no fractures, previous spinal surgery, or interfering spinal anomalies. | 6 cadavers | Intraoperative | X-rays | Segmentation and reconstruction of spines | Deep learning (unspecific) | AI can generate real-time 3D reconstructions from 2D fluoroscopic images of spines, which eliminates the preoperative CT scan and reduces radiation exposure. | This study demonstrated that the X23D-based navigation system for pedicle screw placement shows promise by achieving a low overall breach rate (21% VS. 24%) and requiring less intraoperative ionizing radiation exposure (33.26 mGy VS. 49.47 mGy) compared to the fluoroscopy-aided freehand technique. Limitation: small sample size | This study showed that the X23D-based navigation system for placing pedicle screws works better than the fluoroscopy-assisted freehand technique. It had a lower overall breach rate (21% vs. 24%) and required less ionizing radiation exposure during surgery (33.26 mGy vs. 49.47 mGy). |
| Whole Spine Segmentation Using Object Detection and Semantic Segmentation | Da Mutten, Raffaele, et al. | 2024 | BASE | Retrospective study | Three different data sets from VerSe (spine), MSD T10 (liver), and COVID-19 (chest). | 214 patients for the training set and 40 CT scans for validation | Preoperative | Computed Tomography (CT) | Object detection and segmentation of spines | YOLOv8m and 2D-U-Net | The AI approach improves both detection accuracy and segmentation quality for spines compared to traditional methods. It can reduce the need for manual segmentation and save time for the physician. | The proposed object detection model achieved mAP50-95 of 0.64, 0.63, and 0.09 for training, internal validation, and external validation, respectively. The proposed segmentation model showed a mean Dice score of 0.75± 0.14, 0.76± 0.12, and 0.79± 0.1695 for training, internal validation, and external validation, respectively. | The proposed object detection model (YOLOv8m) achieved mAP50-95 of 0.64, 0.63, and 0.09 for training, internal validation, and external validation, respectively. The proposed segmentation model (2D-Unet) showed a mean DICE score of 0.75 ± 0.14, 0.76 ± 0.12, and 0.79 ± 0.1695 for training, internal validation, and external validation, respectively. |
| SafeRPlan: Safe deep reinforcement learning for intraoperative planning of pedicle screw placement | Ao, Yunke, et al. | 2025 | Pubmed, BASE | Prospective Study | An adult human with a different BMI | 5 adults | Intraoperative | Magnetic resonance imaging (MRI), Computed Tomography (CT), and ultrasound (US) | Registration | SafeRPlan (Deep Reinforcement Learning) | AI achieved 98%–100% safety rates with more than 0.9 insertion ratios, which assists in pedicle screw placement during spinal surgery. It could be integrated into robotic-assisted surgical workflows, improving intraoperative guidance. | SafeRPlan improved safety by 5% compared to existing PSP methods, 99% safety rates  with evaluation on the real US reconstruction dataset. Limitation: lack of data, | It improved safety by 5% compared to existing pedicle screw placement methods, with 99% safety rates, with evaluation on the real US reconstruction dataset. |

**Table S7**: Inter-rater Reliability (Agreement Scores)

| Screening Stage | Total Records Screened | Agreements | Disagreements | Agreement % | Cohen’s Kappa (k) | Interpretation |
| --- | --- | --- | --- | --- | --- | --- |
| Stage 1: Title & Abstract | 1,168 | 1,110 | 58 | 95.0% | 0.89 | Excellent / Almost Perfect |
| Stage 2: Full-Text Review | 91 | 89 | 2 | 97.8% | 0.94 | Excellent / Almost Perfect |

Cohen's Kappa (k) (Scientific Standard)

0.81 – 1.00: Excellent/Almost Perfect

0.61 – 0.80: Substantial/Strong

0.41 – 0.60: Moderate

**Table S8**: Evidence Quality Summary

| **Study design distribution** | **Sample size** | **Setting** | **Cadaveric vs. clinical** |
| --- | --- | --- | --- |
| 5 prospectives,  11 retrospectives | median: 40 patients,  range: 5-282 | 13 single-center,  3 multi-centers | 3 cadaver-only,  11 clinical,  2 mixed |

**References**

1. Faiyazuddin M, Rahman SJQ, Anand G, Siddiqui RK, Mehta R, Khatib MN, et al. The Impact of Artificial Intelligence on Healthcare: A Comprehensive Review of Advancements in Diagnostics, Treatment, and Operational Efficiency. Health Sci Rep. 2025;8(1):e70312.

2. Rasouli JJ, Shao J, Neifert S, Gibbs WN, Habboub G, Steinmetz MP, et al. Artificial intelligence and robotics in spine surgery. Global spine journal. 2021;11(4):556–64.

3. Lee NJ, Lombardi JM, Lehman RA. Artificial intelligence and machine learning applications in spine surgery. International Journal of Spine Surgery. 2023;17(S1):S18–S25.

4. Ibrahim MT, Milliron E, Yu E. Artificial intelligence in spinal imaging-a narrative review. Artificial Intelligence Surgery. 2025;5(1):139–49.

5. Burström G, Buerger C, Hoppenbrouwers J, Nachabe R, Lorenz C, Babic D, et al. Machine learning for automated 3-dimensional segmentation of the spine and suggested placement of pedicle screws based on intraoperative cone-beam computer tomography. Journal of Neurosurgery: Spine. 2019;31(1):147–54.

6. Suganyadevi S, Seethalakshmi V, Balasamy K. A review on deep learning in medical image analysis. International Journal of Multimedia Information Retrieval. 2022;11(1):19–38.

7. Matur AV, Palmisciano P, Duah HO, Chilakapati SS, Cheng JS, Adogwa O. Robotic and navigated pedicle screws are safer and more accurate than fluoroscopic freehand screws: a systematic review and meta-analysis. Spine J. 2023;23(2):197–208.

8. Orief T, Alfawareh M, Halawani M, Attia W, Almusrea K. Accuracy of percutaneous pedicle screw insertion in spinal fixation of traumatic thoracic and lumbar spine fractures. Surg Neurol Int. 2018;9:78.

9. Esfandiari H, Newell R, Anglin C, Street J, Hodgson AJ. A deep learning framework for segmentation and pose estimation of pedicle screw implants based on C-arm fluoroscopy. International journal of computer assisted radiology and surgery. 2018;13:1269–82.

10. Cheng Y, Jiang J-L, Zhang N, Zhao H, Liu Z, editors. Automatic Lumbar Vertebra Landmark Localization and Segmentation for Pedicle Screw Placement. 2022 26th International Conference on Pattern Recognition (ICPR); 2022: IEEE.

11. Thies M, Zäch J-N, Gao C, Taylor R, Navab N, Maier A, et al. A learning-based method for online adjustment of C-arm Cone-beam CT source trajectories for artifact avoidance. International journal of computer assisted radiology and surgery. 2020;15:1787–96.

12. Siemionow KB, Forsthoefel CW, Foy MP, Gawel D, Luciano CJ. Autonomous lumbar spine pedicle screw planning using machine learning: a validation study. Journal of Craniovertebral Junction and Spine. 2021;12(3):223–7.

13. Scherer M, Kausch L, Ishak B, Norajitra T, Vollmuth P, Kiening K, et al. Development and validation of an automated planning tool for navigated lumbosacral pedicle screws using a convolutional neural network. The Spine Journal. 2022;22(10):1666–76.

14. Zhang Q, Zhao F, Zhang Y, Huang M, Gong X, Deng X. Automated measurement of lumbar pedicle screw parameters using deep learning algorithm on preoperative CT scans. Journal of Bone Oncology. 2024;47:100627.

15. Liebmann F, von Atzigen M, Stütz D, Wolf J, Zingg L, Suter D, et al. Automatic registration with continuous pose updates for marker-less surgical navigation in spine surgery. Medical Image Analysis. 2024;91:103027.

16. Yang H-S, Kim K-R, Kim S, Park J-Y. Deep learning application in spinal implant identification. Spine. 2021;46(5):E318–E24.

17. von Atzigen M, Liebmann F, Hoch A, Spirig JM, Farshad M, Snedeker J, et al. Marker-free surgical navigation of rod bending using a stereo neural network and augmented reality in spinal fusion. Medical Image Analysis. 2022;77:102365.

18. Roberts M, Hinton G, Wells AJ, Van Der Veken J, Bajger M, Lee G, et al. Imaging evaluation of a proposed 3D generative model for MRI to CT translation in the lumbar spine. The Spine Journal. 2023;23(11):1602–12.

19. Abel F, Lebl DR, Gorgy G, Dalton D, Chazen JL, Lim E, et al. Deep-learning reconstructed lumbar spine 3D MRI for surgical planning: pedicle screw placement and geometric measurements compared to CT. European Spine Journal. 2024;33(11):4144–54.

20. Luchmann D, Jecklin S, Cavalcanti NA, Laux CJ, Massalimova A, Esfandiari H, et al. Spinal navigation with AI-driven 3D-reconstruction of fluoroscopy images: an ex-vivo feasibility study. BMC Musculoskeletal Disorders. 2024;25(1):925.

21. Da Mutten R, Zanier O, Theiler S, Ryu S-J, Regli L, Serra C, et al. Whole Spine Segmentation Using Object Detection and Semantic Segmentation. Neurospine. 2024;21(1):57.

22. Ao Y, Esfandiari H, Carrillo F, Laux CJ, As Y, Li R, et al. SafeRPlan: Safe deep reinforcement learning for intraoperative planning of pedicle screw placement. Medical Image Analysis. 2025;99:103345.

23. Gertzbein SD, Robbins SE. Accuracy of pedicular screw placement in vivo. Spine. 1990;15(1):11–4.

24. Zdichavsky M, Blauth M, Knop C, Graessner M, Herrmann H, Krettek C, et al. Accuracy of pedicle screw placement in thoracic spine fractures: part I: inter-and intraobserver reliability of the scoring system. European Journal of Trauma. 2004;30(4):234–40.

25. Yu T, Jiao J-H, Wang Y, Wang Q-Y, Jiang W-B, Wang Z-H, et al. Robot-assisted versus navigation-assisted screw placement in spinal vertebrae. International Orthopaedics. 2023;47(2):527–32.

26. Wandvik C, Greil ME, Colby S, Gautam D, Mazur MD. Limitations of current robot-assisted pedicle screw insertion systems. Neurosurgical Focus. 2024;57(6):E14.

27. Liu X, Song L, Liu S, Zhang Y. A review of deep-learning-based medical image segmentation methods. Sustainability. 2021;13(3):1224.

28. Ranjbarzadeh R, Bagherian Kasgari A, Jafarzadeh Ghoushchi S, Anari S, Naseri M, Bendechache M. Brain tumor segmentation based on deep learning and an attention mechanism using MRI multi-modalities brain images. Scientific reports. 2021;11(1):10930.

29. Liu W, Luo J, Yang Y, Wang W, Deng J, Yu L. Automatic lung segmentation in chest X-ray images using improved U-Net. Scientific Reports. 2022;12(1):8649.

30. Yashaswini GN, Manjunath R, Shubha B, Prabha P, Aishwarya N, Manu H. Deep learning technique for automatic liver and liver tumor segmentation in CT images. Journal of Liver Transplantation. 2025;17:100251.

31. Saeed MU, Dikaios N, Dastgir A, Ali G, Hamid M, Hajjej F. An automated deep learning approach for spine segmentation and vertebrae recognition using computed tomography images. Diagnostics. 2023;13(16):2658.

32. Albuquerque C, Henriques R, Castelli M. Deep learning-based object detection algorithms in medical imaging: Systematic review. Heliyon. 2025;11(1).

33. Yeh Y-C, Weng C-H, Huang Y-J, Fu C-J, Tsai T-T, Yeh C-Y. Deep learning approach for automatic landmark detection and alignment analysis in whole-spine lateral radiographs. Scientific reports. 2021;11(1):7618.

34. Islam KT, Wijewickrema S, O’Leary S. A deep learning based framework for the registration of three dimensional multi-modal medical images of the head. Scientific Reports. 2021;11(1):1860.

35. Chen M, Tustison NJ, Jena R, Gee JC. Image registration: Fundamentals and recent advances based on deep learning. Machine Learning for Brain Disorders. 2023:435–58.

36. Gjesteby L, De Man B, Jin Y, Paganetti H, Verburg J, Giantsoudi D, et al. Metal artifact reduction in CT: where are we after four decades? Ieee Access. 2016;4:5826–49.

37. Njiti M, Osman N, Mansor M, Rabaiee N, Aziz MA. Potential of Metal Artifact Reduction (MAR) and Deep Learning-based Reconstruction (DLR) algorithms integration in CT Metal artifact correction: a review. Radiation Physics and Chemistry. 2024;218:111541.
